# *brinker* levels regulated by a promoter proximal element support germ cell homeostasis

**DOI:** 10.1242/dev.199890

**Published:** 2022-02-04

**Authors:** Leslie Dunipace, Susan Newcomb, Angelike Stathopoulos

**Affiliations:** Division of Biology, California Institute of Technology, 1200 East California Boulevard, MC114-96, Pasadena, CA 91125, USA

**Keywords:** *brinker*, Promoter-proximal element, BMP signaling, Oogenesis, Niche, Germline stem cells, Undifferentiated germ cells, *Drosophila melanogaster*

## Abstract

A limited BMP signaling range in the stem cell niche of the ovary protects against germ cell tumors and promotes germ cell homeostasis. The canonical repressor of BMP signaling in both the *Drosophila* embryo and wing disc is the transcription factor Brinker (Brk), yet the expression and potential role of Brk in the germarium has not previously been described. Here, we find that *brk* expression requires a promoter-proximal element (PPE) to support long-distance enhancer action as well as to drive expression in the germarium. Furthermore, PPE subdomains have different activities; in particular, the proximal portion acts as a damper to regulate *brk* levels precisely. Using PPE mutants as well as tissue-specific RNA interference and overexpression, we show that altering *brk* expression within either the soma or the germline affects germ cell homeostasis. Remarkably, we find that Decapentaplegic (Dpp), the main BMP ligand and canonical antagonist of Brk, is upregulated by Brk in the escort cells of the germarium, demonstrating that Brk can positively regulate this pathway.

## Abstract (precis)

**Summary:** A promoter-proximal cis-regulatory element of the gene *brinker* regulates distal enhancer action and also directly acts to control *brinker* expression levels in cells co-expressing BMP ligand thereby influencing germ cell homeostasis.

## INTRODUCTION

**Fig. 1.**
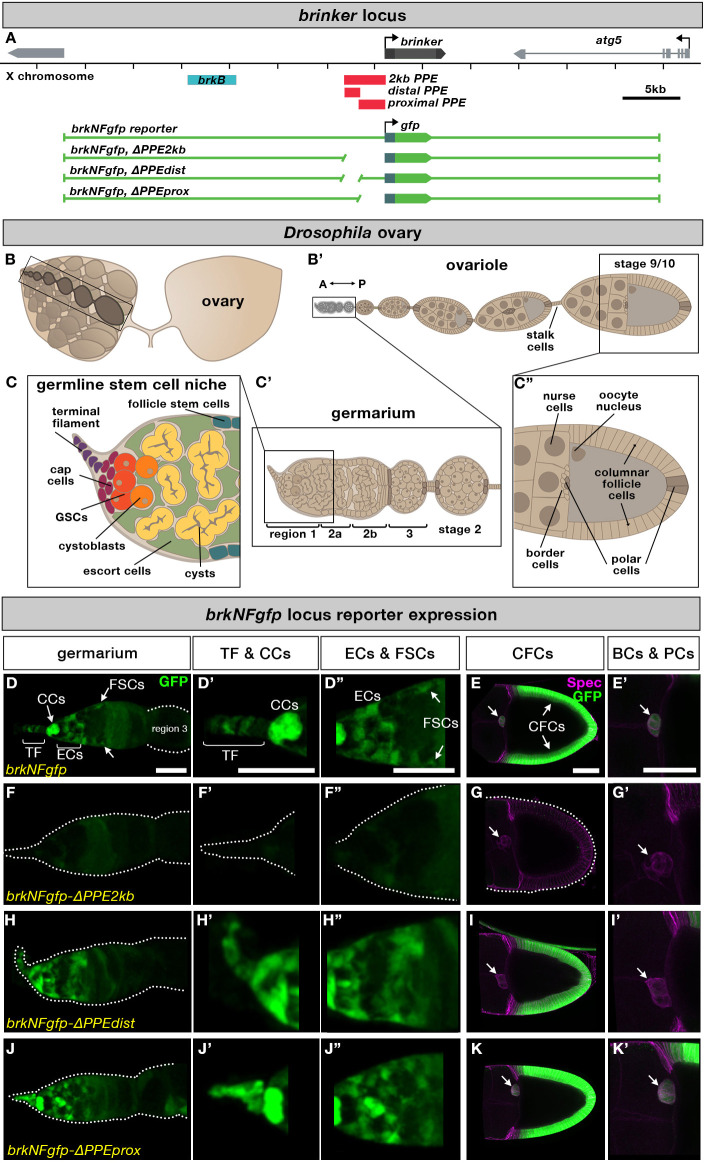
***brk* is expressed in many cell types in the adult ovary and its expression depends on a PPE.** (A) Chromosomal locations of the *brk* gene, PPE (and subunits, red) and *brkB* cis-regulatory module (CRM) (blue). Green line diagrams represent gene regions used in large GFP reporters. (B,B′) Schematic of *Drosophila* ovary and ovariole. (C,C′) Schematics of germline stem cell niche, germarium and posterior half of stage 9/10 follicle. (D-K′) *brk* expression as visualized by *brkNFgfp* shows reporter expression in several somatic cell populations in the germarium (TF, CCs, ECs and FSCs) as well as in developing egg chambers (CFCs, BCs and PCs). Dotted line delineates the outer edge of the ovarian tissue, arrows in E-K indicate the border and follicle cell cluster. Scale bars: 20 μm. In this and all other figures, anterior is to the left.

Maintenance of germline stem cell (GSC) homeostasis is regulated by numerous pathways that signal between the germline and somatic cells that comprise the stem cell niche, as well as by other external and long-range signals (Nelson et al., 2019; Zhang and Cai, 2020). In the *Drosophila melanogaster* model system, the ovary, in which the oocyte develops into a mature egg, contains about fifteen ovarioles composed of germline and somatic cells (Fig. 1B,B′). At the anterior-most tip of each ovariole lies the germarium; a tapered structure made up of several distinct cell types that support the differentiation of one GSC daughter into a cystoblast (CB) and maintenance of the GSC lineage by the other daughter (Fig. 1C,C′). The anterior-most region of the germarium contains the stem cell niche, which comprises three somatic cell populations – the terminal filament (TF), cap cells (CCs) and an anterior subset of escort cells [ECs, alternatively inner germarial sheath (IGS) cells] – and supports the maintenance of two or three GSCs throughout adulthood (Fig. 1C; Liu et al., 2015; Tan et al., 2018; Xie and Spradling, 2000). ECs located more posteriorly influence the differentiation of stem cells, forming what is considered the ‘differentiation niche’ (Kirilly et al., 2011). GSCs produce cystoblasts via asymmetric division aligned along the anterior-posterior axis of the germarium such that daughter cells that move out of the niche escape the self-renewal signal and begin to differentiate, whereas those that remain in contact with the CCs are maintained as GSCs (de Cuevas and Spradling, 1998; Deng and Lin, 1997). This is a complex but well-studied phenomenon that requires the precise localization and interaction of a number of cell signaling pathways (reviewed by Harris and Ashe, 2011; Hayashi et al., 2020; Hsu et al., 2019).

The most pivotal cue is arguably extracellular Decapentaplegic (Dpp), the main *Drosophila* BMP ligand, which is expressed at high levels in and secreted from CCs to promote self-renewal of GSCs within approximately one cell diameter (Eliazer and Buszczak, 2011). In many tissues, Dpp functions as a long-range morphogen to direct cell fate decisions in a concentration-dependent manner across tissues; however, in its role in GSC maintenance, the range of Dpp is limited by the expression of receptors and extracellular matrix components in the niche that serve as a sink for extracellular ligand (Guo and Wang, 2009; Liu et al., 2015; Wang et al., 2008; Wilcockson and Ashe, 2019). This limited signaling range is crucial for proper germline development as GSC division pushes daughter cells out of the range of Dpp and permits differentiation factors, such as the gene *bag of marbles* (*bam*), to be expressed (Song et al., 2004). It was also recently shown that *dpp* is expressed at low levels in ECs to maintain a population of partially differentiated germline cells that can de-differentiate to repopulate the germarium (Liu et al., 2015); a result suggesting that not only the presence of signals, but also their expression levels, can be interpreted by the germline to affect cell fate.

The transcription factor Brinker (Brk) encodes a canonical repressor of Bmp signaling and has been demonstrated to repress expression of both *dpp* (Hasson et al., 2001; Theisen et al., 2007) and Dpp-dependent target genes (Rushlow et al., 2001; Sivasankaran et al., 2000). Inversely, BMP signaling activates a complex that directly represses expression of *brk* (Marty et al., 2000). As a result of this mutual repression, *brk* is typically expressed in an obverse pattern to *dpp*, for example in the embryo (Jaźwińska et al., 1999a) and wing imaginal disc (Campbell and Tomlinson, 1999; Minami et al., 1999), which, in addition to other mechanisms, helps shape the Dpp gradient (Affolter et al., 2001; Müller et al., 2003; O'Connor et al., 2006). Brk acts similarly in the ovaries starting at stage 8 of oogenesis when it is important for establishing the anterior-posterior gradient of Dpp expression, which patterns the eggshell and is essential for dorsal appendage and operculum formation (Chen and Schüpbach, 2006). Despite extensive studies of Dpp expression in the germarium, however, the role of Brk in this tissue has not been previously described.

Here, we show that, not only is *brk* expressed in the germarium, but its expression coincides with and, remarkably, positively regulates *dpp* expression in somatic cells. We also demonstrate that a previously described promoter-proximal element (PPE), first characterized as supporting distal enhancer action in the early embryo (Dunipace et al., 2013), also has a role in the ovary.

## RESULTS

### The *brk* PPE regulates expression in multiple ovarian tissues and is required for distal enhancer action

To examine the role of the PPE in supporting ovary *brk* expression, we first used a set of large reporters in which the *brk* coding sequence is replaced with *gfp* in the context of ∼30 kb of flanking sequence (*brkNFgfp*). We examined wild-type reporter expression as well as that of reporters with deletions of the full-length PPE (*PPE2kb*) or its distal or proximal subdomains (*PPEdist* and *PPEprox*, respectively) (Fig. 1A; Dunipace et al., 2013). These reporters were used previously in the embryo to show that the PPE does not itself drive expression but instead serves to facilitate the action of other enhancers located at a distance (Dunipace et al., 2013). To identify the cell types expressing *brk* reporters, ovaries were co-stained with antibodies for Traffic jam (Tj) to mark all follicle cells except for the TFs, Lamin C (LamC) to mark CCs and TF, and/or α-Spectrin (Spec), which outlines all later-stage follicle cells as well as marking both spectrosomes (a specialized rounded organelle found in GSCs and cystoblasts) and fusomes (found on differentiating cysts) (de Cuevas et al., 1996; Li et al., 2003; Xie and Spradling, 2000). In the germarium, *brkNFgfp* was expressed in a number of cell types: TF, CCs, ECs and follicle stem cells (FSCs) (Fig. 1D-D″). Expression persisted in follicle cells throughout egg chamber development (Fig. S1A), becoming restricted after stage 7 to only the columnar follicle cells (CFCs) and the polar and border follicle cells (PCs, BCs) (Fig. 1C″,E,E′). Deletion of *PPE2kb* from *brkNFgfp* (*brkNFgfp-*Δ*PPE2kb*) abolished GFP reporter expression in the ovary, except for some low level expression in the FSC region (Fig. 1F-G′), indicating that the PPE is required for all *brk* expression in this tissue.

Previously, we found that the PPE could be divided into subunits that were largely redundant in their ability to support distal enhancer action in the early embryo (Dunipace et al., 2013). We therefore examined expression of the *brkNFgfp* reporter in which either the distal or proximal PPE domain is deleted (*brkNFgfp-*Δ*PPEdist* and *brkNFgfp-*Δ*PPEprox*, respectively). In the germarium, *PPEdist* and *PPEprox* appeared to be generally redundant in their ability to support *brkNFgfp* reporter expression (Fig. 1H-H″,J-J″). Similarly, each domain appeared to be sufficient to support CFC reporter expression (Fig. 1I,K). However, in *brkNFgfp-*Δ*PPEdist* specifically, GFP signal was lost in many follicle cells associated with mid-stage egg chambers (Fig. S1B), including stage 10 BCs/PCs (Fig. 1I′), indicating a distinct requirement for that element to support expression in a subset of cells.

**Fig. 2.**
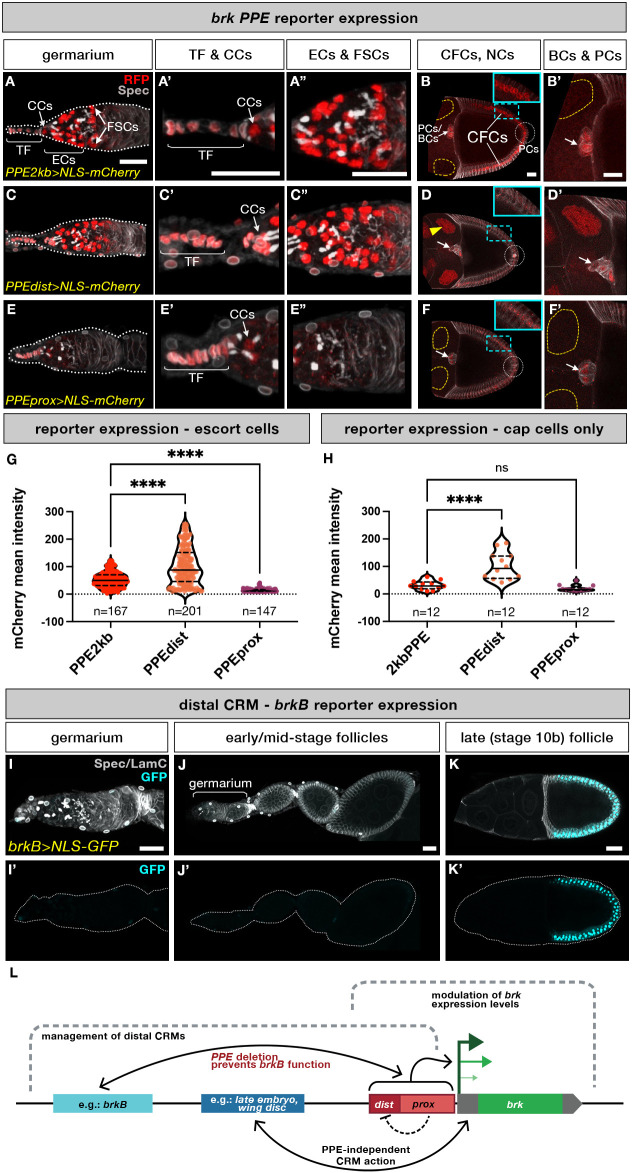
***brk* PPE drives expression in the germarium and is required to facilitate expression of distal CRMs in later-stage egg-chambers*.*** (A-F′) Transgenic reporters of *brk* PPE and its distal and proximal subdomains driving nuclear mCherry show expression in subsets of *brk*-expressing cell types. White arrows indicate BC/PC cluster, white dotted circle indicates posterior PC region. Yellow arrowhead indicates nurse cell nuclei that express the reporter whereas nurse cell nuclei that do not express reporter are outlined in yellow. Blue dashed rectangles indicate the area shown in the insets (B,D,F). (G,H) Quantification of PPE reporter expression levels in escort cells of the germarium (G) or in cap cells (H). One-way ANOVA was used for statistical comparison of each dataset to Δ*PPE2kb* (see Materials and Methods). *n*=number of nuclei error bars represent mean±s.d. (see Table S2). (I-K′) Expression of nuclear *brkB-GFP* reporter in the ovariole. Dotted lines delineate the outer edge of the ovarian tissue. Scale bars: 20 µm. (L) Schematic illustrating the bimodal role of the PPE in supporting CRM-driven *brk* expression in the ovary (not drawn to scale).

In order to explore further the sufficiency of the PPE to drive expression in the ovary, we created direct-fusion nuclear-localized RFP transgenic reporters of the PPE and its distal and proximal subdomains (*PPE2kb>*, *PPEdist>* and *PPEprox>NLS-mCherry*, respectively; Fig. 1A; Table S1; see Materials and Methods). These small transgenic reporters revealed that the PPE acts as a traditional enhancer in the ovary as it is capable of driving expression in multiple cell types (Fig. 2A-F′). The *PPE2kb* reporter is active in most of the somatic tissues in the niche: the TF, CCs and ECs (Fig. 2A-A″), but was not detectably expressed in the germline, either in the germarium or in later-stage nurse cells (Fig. 2A,B). In stage 10 egg chambers, *PPE2kb* drove expression in the BCs and PCs but only weakly in the CFCs (Fig. 2B,B′).

When we examined direct reporter expression driven by PPE subdomains, we observed significant differences in levels of activity. The nuclear localization of these reporters permitted quantification of differences in levels of expression that were not readily observable from the cytoplasmic *brkNFgfp* signal, especially in ECs because these cells form extensive protrusions into the germline (Kirilly et al., 2011). Expression levels of small reporters were quantified in accessible cell types, the ECs and CCs in the germarium, but excluding TFs, which were variable as a result of mounting (Fig. 2G,H; Table S2; see Materials and Methods). In the germarium, *PPEdist* drove higher levels of expression in CCs and ECs than the full *PPE2kb* (Fig. 2C-C″,G,H). Inversely, *PPEprox* drove low level expression in CCs and a subset of ECs (Fig. 2E-E″,G,H). In late-stage egg chambers, *PPEdist* was expressed in both BCs and PCs, but drove little to no CFC expression (Fig. 2D,D′). By contrast, *PPEprox* supported weak expression in BC and CFCs, but did not support expression in PCs (Fig. 2F,F′). Finally, *PPEdist* was active in the late-stage germline where *brkNFgfp* and other *brk* reporters were not detected (Fig. 2D,D′; Fig. S1E). These results indicate that both halves of the PPE can drive reporter expression in both CCs and ECs in the germarium, but at different levels (distal high, proximal low) and that wild-type expression levels (i.e. those of the full-length *PPE2kb*) require both halves. In late-stage egg chambers, the two PPE domains act more additively with each supporting subsets of the full expression pattern, except in the case of the germline expression of *PPEdist*, which is repressed in the context of the full *PPE2kb*.

To provide insight into PPE function in CFCs, we examined a previously described distal *brk* cis-regulatory module (CRM), *brkB* (Charbonnier et al., 2015), which is not active in the germarium or mid-stage egg chambers (Fig. 2I-J′) but does drive strong expression exclusively in the CFCs (Fig. 2K,K′). The fact that the PPE itself is not a strong CFC driver (Fig. 2B), but *brkNFgfp* reporter expression is lost upon PPE deletion (Fig. 1G), indicates that the PPE is required for *brkB* activity in CFCs. Also, like the redundancy previously noted in the early embryo, neither distal nor proximal deletion affects PPE reporter expression in CFCs (Fig. 1I,K), indicating that either region is sufficient to support the action of distal enhancers, such as *brkB*. Taken together, this reporter analysis suggests that in the ovary the PPE has two functions: to facilitate the action of other enhancers and to serve as a direct driver of *brk* expression (Fig. 2L).

### *brk* PPE supports maintenance of germline homeostasis

**Fig. 3.**
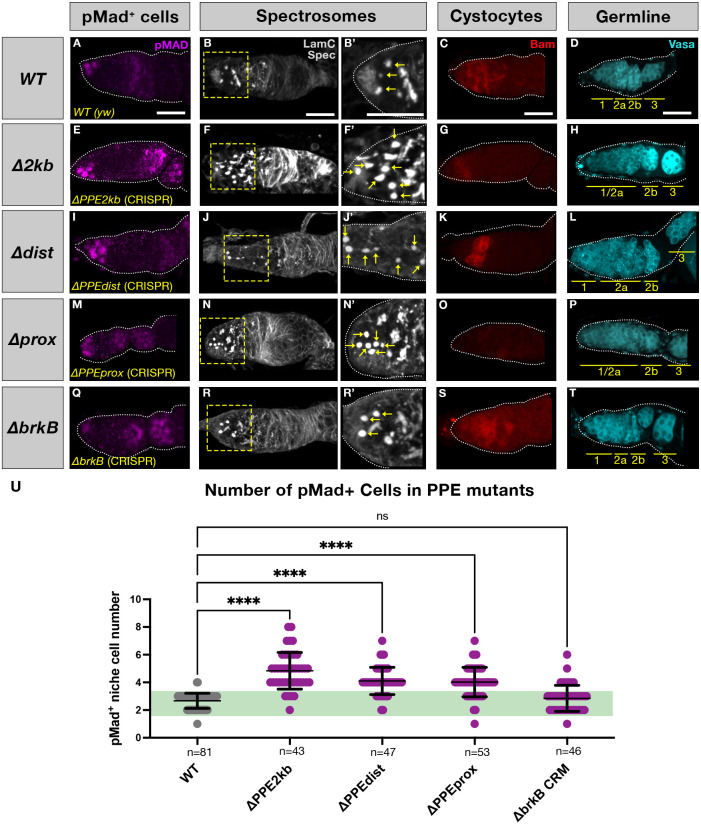
***brk* PPE mutants affect undifferentiated germline cell number.** Representative images for germaria of genotypes WT (A-D), Δ*PPE2kb* (E-H), Δ*PPEdist* (I-L), Δ*PPEprox* (M-P) and Δ*brkB* (Q-T) stained for pMad (magenta), Spectrin (gray), Bam (red) and Vasa (cyan). Yellow dashed boxes indicate the areas shown at higher magnification. Yellow arrows indicate rounded spectrosomes. Yellow bars indicate germarium regions. Scale bars: 20 μm. (U) pMad-positive niche cells (i.e. pMad^+^ cells) present in region 1 (see Fig. 1C) were counted for each genotype; green band delineates the ‘normal’ range of two or three pMad^+^ cells per germarium. One-way ANOVA was used for statistical comparison of each dataset to WT (see Materials and Methods). *n*=number of germaria; error bars represent mean±s.d. (see Table S2).

The fact that we observe *brk* expression in cells that comprise the germline stem cell niche (i.e. TF, CCs and ECs) suggests that Brk plays a role in regulating germline homeostasis. To test this, we generated deletions of the PPE and its distal and proximal subdomains in the context of the endogenous *brk* locus using CRISPR-Cas9 genome editing (Δ*PPE2kb*, Δ*PPEdist* and Δ*PPEprox*, respectively; see Materials and Methods, Tables S1 and S3). We also deleted *brkB* (Charbonnier et al., 2015) in the same manner (Δ*brkB*)*.* PPE, but not *brkB*, deletions had significant effects on germarium morphology, including germline differentiation, spectrosome number and distribution, and the overall organization of the germline, as well as expression pattern and level of Bam, which marks differentiating cystoblasts (Fig. 3). Specifically, whereas wild-type germaria contained two or three GSCs, which present rounded spectrosomes, contact the CCs, and can be labeled by phosphorylated Mothers Against Decapentaplegic (pMad) antibody staining (Fig. 3A,U; Song et al., 2004), germaria from PPE mutant females consistently contained significantly more pMad^+^ cells (Fig. 3E,I,M,U). This pMad^+^ cell population likely contains true GSCs (in contact with CCs) as well as dysregulated cystoblasts (located in proximity to, but not directly contacting the CCs) and will be referred to collectively hereafter as pMad^+^ cells. Counterintuitively, this increase in pMad^+^ cell number occurred in all PPE mutants (i.e. Δ*PPE2kb*, Δ*PPEdist* and Δ*PPEprox*), despite the fact that these deletions had varying effects on *brk* reporter expression levels (Fig. 2G,H). Furthermore, pMad^+^ cell number was unaffected in Δ*brkB* germaria (Fig. 3Q,U), indicating that this is a PPE-specific effect. We also observed that the number of rounded spectrosomes, which mark GSCs and cystoblasts, increased correspondingly with pMad^+^ cell number, confirming that these changes represent a delay in the differentiation of pMad^+^ cells and cystoblasts into more mature cysts (Fig. 3B,B′,F,F′,J,J′,N,N′,R,R′).

These observations of effects on germline homeostasis in the niche are further supported by our findings of corresponding changes in overall morphology of the germline in mutant germaria. The germline in wild-type germaria can be divided into morphological regions whereby region 1 contains the GSCs, cystoblasts and 2- to 8-cell cysts; region 2a contains the 16-cell cysts and 2b the same cysts once they have adopted an elongated, lens-like shape that spans the width of the germarium; and region 3 contains a spherical cluster comprising 15 nurse cells and one oocyte completely enclosed by follicle cells (see Fig. 1C′; King, 1970; McKearin and Ohlstein, 1995). PPE mutants showed aberrant germline morphology with tumorous expansion of regions 1 and 2a with region 2b sometimes affected (Fig. 3H,L,P compared with 3D). Cyst organization appeared to recover by region 3, which was structured normally in nearly all samples. Germline organization appeared normal in Δ*brkB* germaria (Fig. 3T). Strikingly, Δ*PPEprox* ovaries also lacked detectable Bam expression (Fig. 3O) whereas Bam was present in all other genotypes examined (Fig. 3C,G,K,S). This loss of the key differentiation factor, Bam, is likely to be a contributor to the elongated region 1 and lack of distinction of region 2 of Δ*PPEprox* germaria. However, the severity of this particular germline phenotype (i.e. loss of Bam) did not correlate with fertility: Δ*PPEdist* and Δ*PPEprox* mutants are both viable and fertile whereas Δ*PPE2kb* mutants are semi-lethal (few survivors) and female sterile, and the *brkB* mutants are healthy but female sterile. Because Δ*PPEprox* mutants are normally fertile, it is likely that the lack of detectable Bam is rescued by a counteracting effect on the gene network, such as repression of Pumillio (Pum) as *pum*, *bam* double mutants have been shown to rescue differentiation defects caused by loss of Bam alone (Chen and McKearin, 2005).

### In the germarium, *brk* is expressed in both the germline and soma and its expression level is correlated with that of *dpp*, which encodes its canonical antagonist

**Fig. 4.**
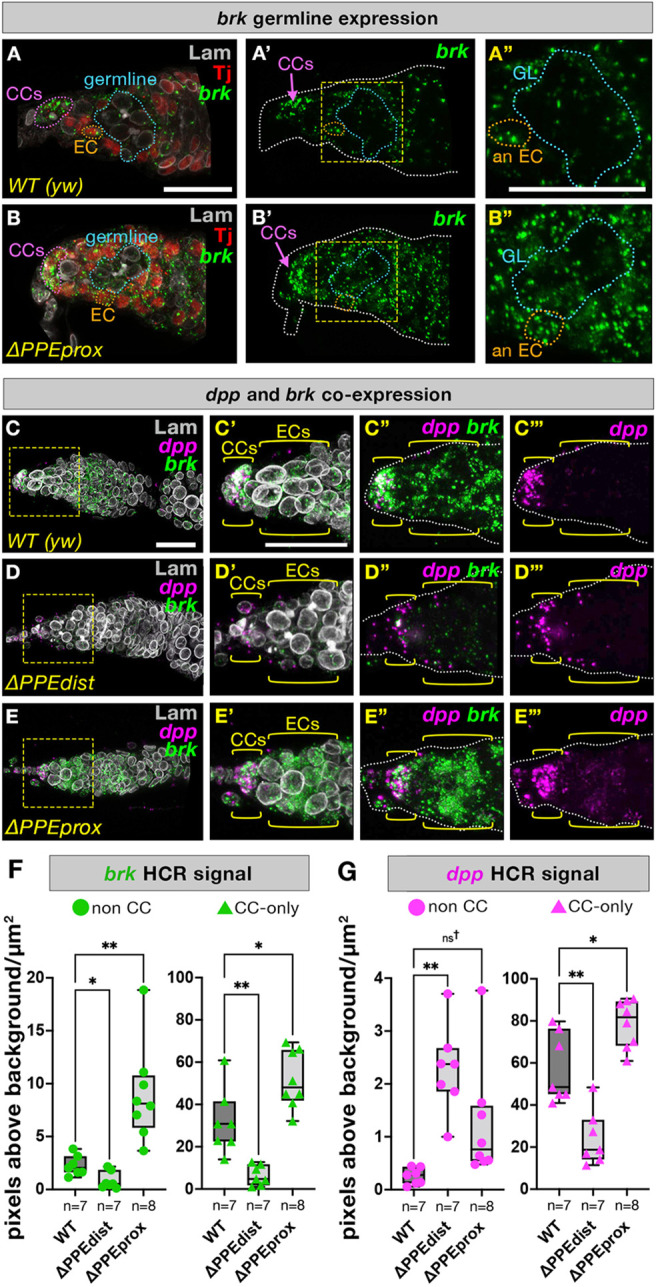
***brk* is expressed in the germline as well as colocalizing and correlating with *dpp* expression in soma.** (A-B″) HCR against *brk* in the niche region of germaria in WT (A-A″) and Δ*PPEprox* mutants (B-B″). Follicle cells are stained with Tj antibody (red) and all nuclei are outlined with Lam (white). CCs and representative ECs are outlined in magenta and orange, respectively. Germline regions are outlined in blue and marked by the absence of red Tj staining. Yellow dashed boxes indicate the areas shown at higher magnification. (C-G) HCR colocalization of *brk* and *dpp* was assessed in the niche region of WT (C-C‴), Δ*PPEdist* (D-D‴) and Δ*PPEprox* (E-E‴) germaria and quantified in whole germaria excluding CCs (F) and in CCs alone (G) by measuring pixel intensity above threshold normalized by area (see Materials and Methods). One-way ANOVA was used for statistical comparison of each dataset to WT (see Materials and Methods). Dagger symbol indicates that these genotypes were additionally compared by xxxxxxxxx *t*-test, which identified a statistically significant difference (*P*=0.0307, see Materials and Methods). *n*=number of germaria; box plots extend from the 25th to 75th percentiles with the horizontal line indicating the median and with whiskers indicating minimum and maximum values (see Table S2). Scale bars: 20 µm.

Our analysis of *brk* PPE reporters suggest a model where *PPEprox* functions to downregulate *brk* gene expression as *PPEdist* alone is a stronger driver of reporter expression than the full-length *PPE2kb* (Fig. 2G,H). To confirm and quantify *brk* expression in wild-type and PPE deletions, we used *in situ* hybridization chain reaction (HCR), which relies on signal amplification to permit highly sensitive labeling of RNA in fixed samples (Choi et al., 2018). We observed colocalization of *brk* HCR signal with Tj antibody staining, confirming expression of *brk* in all ECs and CCs in both wild-type and Δ*PPEprox* mutant germaria (Fig. 4A,B). Our analysis of the *PPEdist-mCherry* reporter raised the possibility that *brk* might be expressed in the germline (Fig. 2D); however, reporters have been previously shown to be regulated in unexpected ways specifically in the *Drosophila* germline (DeLuca and Spradling, 2018). We therefore used HCR to examine *brk* expression in the germline. Because HCR signal is non-nuclear, assigning cell identity to observed spots is challenging in the germarium, where ECs form projections that surround germline cells (Kirilly et al., 2011). To overcome this, we examined confocal stacks of germaria co-stained with Tj antibody and did indeed observe *brk* expression in the germline, i.e. only those cells in the center of germaria that lack Tj staining (Fig. 4A-A″, blue dotted outline). This germline *brk* expression was upregulated in Δ*PPEprox* mutants (Fig. 4B-B″, compare with 4A-A″). We quantified *brk* expression levels in the early germarium as a whole (stages 1-2a, follicle and germline cells representing ‘non-CCs’), as well as in CCs alone by counting pixels above background normalized to area in wild type as well as in Δ*PPEdist* and Δ*PPEprox* mutants (see Materials and Methods). Consistent with the trends observed from the H2A-mCherry reporter constructs, this HCR analysis confirmed that *brk* expression is lower in Δ*PPEdist* mutants and upregulated in Δ*PPEprox* mutants in both the CC and non-CC regions of the germarium (Fig. 4F). Taken together, both reporter and HCR results support a model in which *PPEprox* acts as a ‘damper’ to downregulate expression supported by *PPEdist* in all *brk*-expressing cell types of the germarium.

We had previously noted that *brk* PPE mutants have altered numbers of pMad^+^ cells and reasoned that this might be caused by changing expression of the BMP ligand Dpp, which provides a key self-renewal signal for GSCs and has a well-studied repressive regulatory relationship with *brk*. Again using HCR, we co-stained for *brk* and *dpp* transcripts in wild-type and PPE mutant ovarioles (Fig. 4C-E; Fig. S2A-D″). Surprisingly, we found that *brk* and *dpp* expression domains overlap in wild-type germaria, with *dpp* and *brk* co-expressed at high levels in CCs, as well as at lower levels throughout the germarium (Fig. 4C-C‴). Although this was an unexpected result (e.g. Jaźwińska et al., 1999b; Minami et al., 1999), our observation of co-expression of *brk* and *dpp* in the germarium is supported by two recent single-cell RNA-sequencing studies of the ovary (Rust et al., 2020; Slaidina et al., 2020). We also observed a corresponding increase in *dpp* levels in CCs as well as non-CC cell types in Δ*PPEprox* germaria (Fig. 4E-E‴,G) indicating that, in contrast to its canonical role as a repressor of *dpp* and its target genes (e.g. Jaźwińska et al., 1999a; Takaesu et al., 2008; Theisen et al., 2007), Brk upregulates *dpp* expression in this developmental context. In keeping with this trend, loss of *brk* in CCs of Δ*PPEdist* germaria led to loss of *dpp* expression, again indicating a positive regulatory relationship (Fig. 4D-D‴,G). In contrast, however, we observed an increase in *dpp* expression in non-CC cells of Δ*PPEdist* germaria despite a loss of *brk* in that region. Although this finding of increased non-CC *dpp* in Δ*PPEdist* mutants helps clarify the contradictory observation that all PPE mutants have increased pMad^+^ cell number despite varying effects on *brk* levels (Fig. 3U), these experiments do not shed light on why we did not observe a positive *brk-dpp* regulatory relationship in the context of the Δ*PPEdist* non-CCs*.* One explanation is that, because these are constitutive PPE mutants, they may have effects in multiple interacting cell types (including within the germline; see below) or at earlier time points.

Importantly, the positive relationship between *brk* and *dpp* observed in Δ*PPEprox* mutants and in the CCs of Δ*PPEdist* mutants is specific to the germarium, as these genes exhibited an obverse expression pattern in late-stage egg chambers, as would be expected from their canonical mutually repressive relationship (Fig. S2A-B″).

### Precise regulation of *brk* expression level in both soma and germline is required for germ cell differentiation

**Fig. 5.**
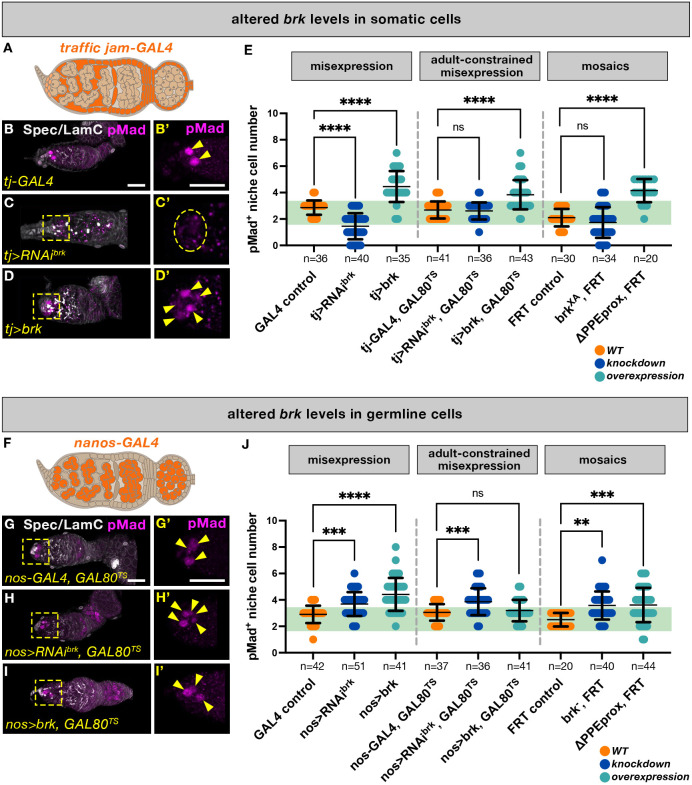
**Upregulation of *brk* in somatic tissues or downregulation of *brk* in the germline both lead to increased pMad^+^ cells.** (A,F) Schematics of expression patterns of *tj-GAL4* (A) and *nos-GAL4* (F) drivers used to affect *brk* expression in escort and follicle cells and in the germline, respectively. (B-D′,G-I′) Maximum projections of representative germaria from somatic experiments of WT flies (B,B′) and those in which *tj-GAL4* was used to drive either *brk* RNAi (C,C′) or overexpression (D,D′), or germline experiments of representative germaria from WT flies (G,G′) and those in which *nos-GAL4* was used to drive either *brk* RNAi (H,H′) or overexpression (I,I′) in the background of GAL80^TS^ to constrain GAL4 activity to the adult stage. Germaria were stained with anti-Spectrin, anti-Lamin C (gray) and anti-pMad (magenta) antibodies. Yellow dashed boxes indicate inset area, yellow arrowheads indicate individual pMad^+^ cells, yellow dashed oval indicates niche region lacking pMad^+^ cells. Scale bars: 20 μm. (E,J) Quantification of pMad^+^ niche cells in *brk* misexpression experiments, with and without GAL80^TS^, and in *tj-GAL4*- (E) and *nos-GAL4*- (J) driven cell type-specific mosaics of either *brk* mutant alleles or of the proximal PPE deletion mutant. pMad^+^ cells were quantified for each genotype by counting the number of pMad^+^ cells in the niche region; green band signifies ‘normal’ range of two or three pMad^+^ cells per germarium. One-way ANOVA was used for statistical comparison of each dataset to its respective control genotype (see Materials and Methods). *n*=number of germaria; error bars represent mean±s.d. (see Table S2).

As the germarium phenotypes described above (Fig. 3) resulted from constitutive PPE mutants, we next sought to identify specific tissues in which regulation of *brk* levels contributes to germline homeostasis. We used two approaches based on the GAL4/UAS binary expression system to manipulate *brk* levels and to generate cell type-specific mutants (Brand and Perrimon, 1993; Duffy et al., 1998). Using a GAL4 driver active in either the soma (all follicle cells except TF and stalk cells), *tj-GAL4* (Fig. 5A; Sahai-Hernandez and Nystul, 2013), or the germline, *nos-GAL4* (Fig. 5F; Van Doren et al., 1998), we first performed tissue-specific *brk* overexpression and RNA interference (RNAi). We repeated these misexpression experiments in the presence of temperature-sensitive GAL80 (*GAL80^TS^*) and with timed temperature shifts to constrain GAL4 activity to the adult, as constitutive mutants might produce effects at earlier developmental time points that could confound interpretation of phenotypes (McGuire et al., 2004; see Materials and Methods).

Second, we used *brk* null mutants with linked FRT sites to facilitate generation of cell type-specific *brk^−^* mosaic clones using the same GAL4 drivers described above. In short, GAL4 expression in the presence of UAS-FLP recombinase drives mitotic recombination between *brk* mutant, FRT chromosomes and a wild-type FRT homolog generating mosaics only in the GAL4-expressing tissue (see Materials and Methods). These null clones served to validate our observations from *brk* RNAi and as a proxy for *brk*^Δ*PPE2kb*^ and *brk*^Δ*PPEdist*^, as both these lesions functioned as mutants in that they led to a reduction in *brk* reporter expression across all germarium cell types (Figs 1F and 4F). We also generated corresponding Δ*PPEprox*, FRT recombinants to make cell type-specific *brk*^Δ*PPEprox*^ mosaic clones, which we predicted would cause clonal *brk* overexpression for comparison with parallel *UAS-brk* experiments.

Because we observed *dpp* upregulation as a result of increased *brk* in Δ*PPEprox* mutants and *dpp* is a key GSC maintenance signal, we reasoned that this phenomenon might explain the increased pMad^+^ cell phenotype observed in this genotype. We therefore first examined germaria in which *brk* was overexpressed in specific cell types. Indeed, somatic overexpression of *brk* using either *UAS-brk* or *brk*^Δ*PPEprox*^-FRT with *tj-GAL4* led to an increase in pMad^+^ cell number (Fig. 5D,E). Parallel adult-constrained overexpression experiments with *GAL80^TS^* (see Materials and Methods) were not significantly different from non-*GAL80* experiments, indicating that pupal *tj-GAL4* activity does not contribute to the increased pMad^+^ cell number in the case of somatic *brk* ectopic expression (Fig. 5E). The *tj-GAL4* driver was expressed in most follicle cells of the ovary, including weakly in CCs and stronger in all ECs, with stronger expression in the follicle cells associated with later-stage egg chambers (Fig. 5A; Li et al., 2003). In order to identify specific somatic domains in which *brk* overexpression affects pMad^+^ cell number, we next performed parallel overexpression experiments using additional GAL4 drivers active in subsets of follicle cells: *c587-GAL4*, a strong driver in all anterior follicle cells of the germarium except CCs (Jin et al., 2013), and *GMR25A11-GAL4*, a strong driver in a subset of region 1 and 2a ECs (Liu et al., 2015). As expected by their largely overlapping expression profiles in the anterior germarium, *C587-*driven, adult-constrained *brk* overexpression recapitulated *tj-GAL4* overexpression (Fig. S3A). In addition, there was also a significant increase in pMad^+^ cell number when *brk* was overexpressed only in a subset of anterior ECs using *GMR25A11-GAL4* (Fig. S3B). As the anterior ECs represent the intersection of these three drivers' expression domains, with *GMR25A11* being highly specific to that cell type, these data collectively support the conclusion that increased *brk* in anterior ECs is sufficient to induce an increase in pMad^+^ cell number. In contrast, *brk* overexpression experiments using a driver specific to FSCs and later follicle cells, *109-30-GAL4* (Hartman et al., 2015), did not lead to an increase in pMad^+^ cells (Fig. S3C). Taken together, these overexpression experiments establish that increased *brk* levels in anterior ECs alone is sufficient to induce an increase in pMad^+^ cell number.

In parallel, these drivers were also used to assess the effects of loss of *brk* in somatic cells using *brk* RNAi or by generating tissue-specific *brk* null clones. In general, loss of *brk* in the somatic cells led to a loss of pMad^+^ cells, the opposite effect to that observed in overexpression experiments (Fig. 5C,E; Fig. S3A,B). Additionally, *tj>brkRNAi* ovaries often had collapsed germaria containing only one or no pMad^+^ cells and many spectrosomes (Fig. 5C). There was, however, no significant change in pMad^+^ cell number in either adult-constrained *tj-GAL4* knockdown or in *brk* mutant mosaics using this driver (Fig. 5E), indicating that loss of somatic *brk* at an earlier stage in development may contribute to the low pMad^+^ cell number phenotype and the collapse of the differentiation niche. However, adult-constrained *c587-GAL4* knockdown experiments did show a decrease in pMad^+^ cell number (Fig. S3A), indicating that adult knockdown of *brk* can also affect pMad^+^ cell number. These germaria also have more spectrosomes, similar to *tj>brkRNAi* experiments (Fig. S4B; compare with Fig. 5C). The discrepancies between *tj* and *c587* adult-constrained experiments could be explained by the differences in the subsets of germarium cell types that express these drivers (i.e. TF by *C587-GAL4* and CCs by *tj-GAL4*), but may also be due to a difference in driver strength. Indeed, when we compared *c587-* or *tj-*driven *UAS-mcd8-GFP* reporter expression, GFP signal was markedly higher in *c587-GAL4* germaria (Fig. S4E,F). This difference in levels correlates with the severity of collective germarium phenotypes, with the weaker driver, *tj-GAL4*, producing perturbed germaria but a wild-type number of pMad^+^ cells, and the stronger driver, *c587-GAL4*, causing more severely perturbed germaria and a reduction or complete loss of pMad^+^ cells (Fig. S4A,B). The fact that experiments with a third somatic driver expressed in ECs, *GMR25A11-GAL4*, could produce either an increase or a decrease in pMad^+^ cell number from somatic *brk* overexpression or knockdown, respectively (Fig. S3B), lends further support to the conclusion that changes in expression of *brk* – in either direction – specifically in anterior ECs is sufficient to affect pMad^+^ cell number.

We again performed parallel experiments using an alternative somatic driver, *109-30-GAL4*, which is specific to FSCs and later follicle cells (Hartman et al., 2015). Although experiments with this driver did not significantly affect germ cell number, except in Δ*PPEprox* mosaics (Fig. S3C), *109-30*-driven *brk* knockdown did result in a significant reduction in the number of mid-stage egg chambers (Fig. S4C). This loss of mid-stage egg chambers was also observed after adult-constrained *brk* knockdown with either *tj-GAL4* or *c587-GAL4*, both of which are active in FSCs (Fig. S4A,B), but not with *GMR25A11-GAL4*, which is not expressed in the FSCs (Fig. S4D). To quantify this phenomenon, we counted the number of egg chambers per ovariole for each of these *brk* knockdown genotypes. All drivers active in later FCs (*tj-GAL4*, *c587-GAL4* and *109-30-GAL4*) showed a significant reduction in egg chamber number, but the early EC driver (*GMR25A11-GAL4*), which is absent from FSCs and later FCs, did not (Fig. S4G). Taken together, these results indicate that Brk is required in FSCs and later-stage follicle cells for specification and/or proliferation of follicle cells outside the niche. The lack of effect on germ cell number in *109-30-GAL4* and adult-constrained *tj-GAL4 brk* RNAi experiments further suggests that this FSC role is separable from the contribution of Brk to germline differentiation.

Because both the *tj-GAL4* and *c587-GAL4* experiments produced significant changes in pMad^+^ cell number and both drivers showed some expression in either the CCs or TF, tissues that are known to contribute to germline homeostasis, we sought to identify whether *brk* expression levels in these tissues might also affect pMad^+^ cell number. To investigate this possibility, we performed *brk* overexpression and knockdown experiments using the *bab1-GAL4* driver (constrained to the adult with *GAL80^TS^*), which is specific to TF and CCs (Bolívar et al., 2006). Although *bab1*-driven *brk* overexpression did result in a significant increase in pMad^+^ cells, in agreement with other somatic driver experiments, parallel *bab1*-driven *brk* knockdown produced the same phenotype (Fig. S3D). This finding conflicts with results from all other anterior somatic drivers tested (Fig. 5E; Fig. S3A,B). One possible explanation is that our use of *GAL80^TS^* was not sufficient to constrain *bab1* activity to the adult and our observations were therefore confounded by leaky pupal GAL4 activity. Alternatively, *brk* levels in the TF and CCs may have different effects that conflict when *brk* is either up- or downregulated in both cell populations. However, further testing for a specific requirement for *brk* in the TF and CCs was not possible because FRT experiments are not appropriate for this driver, as they depend on mitosis to generate clones and TF and CCs do not divide in the adult (Bolívar et al., 2006). Although our HCR imaging suggests that constitutive ectopic *brk* does indeed lead to an increase in *dpp* specifically in CCs (Fig. 4G), we were not able to discern the contribution of CC-specific *brk* expression to germ cell differentiation.

In addition to its role in the soma, *brk* expression in the germline itself is also required for proper germ cell differentiation. Despite the fact that *brk* expression was not detectable in the germline using either the large (*brkNFgfp*) or small (*PPE2kb-mCherry*) reporter (Figs 1D and 2A), loss of germline *brk* (either misexpression or mosaic) generated using the germline-specific *nos-GAL4* driver (Fig. 5F) led to increased GSC number (Fig. 5G-J). These results add support to our finding that *brk* is expressed in the germline in wild-type germaria and further suggest that its presence there is important to support germ cell homeostasis. In experiments without *GAL80^TS^*, this effect was also observed in overexpression conditions (*nos>brk* and Δ*PPEprox, FRT*; Fig. 5J); however, addition of *GAL80^TS^* and appropriate temperature shifts rescued this phenotype, indicating that pupal *nos-GAL4* activity was responsible for the overexpression-pMad^+^ cell loss phenotype (Fig. 5J). Furthermore, experiments using *bam-GAL4*, which is active only in the cystoblast and the 2-8 cell cysts (Chen and McKearin, 2003), recapitulated the trends in pMad^+^ cell number observed in *nos-GAL4, GAL80^TS^* experiments (Fig. S3E). This result underscores the important role that *brk* plays in the germline despite its relatively low levels of expression there, as knocking down *brk* even in very few cells significantly impacts germ cell differentiation. Unlike the experiments described above that alter expression in somatic cells, changing *brk* levels in the germline does not have noticeable effects on fertility or strong defects in later-stage egg chambers, indicating that germline *brk* expression is important for GSC homeostasis but not for later ovariole development.

**Fig. 6.**
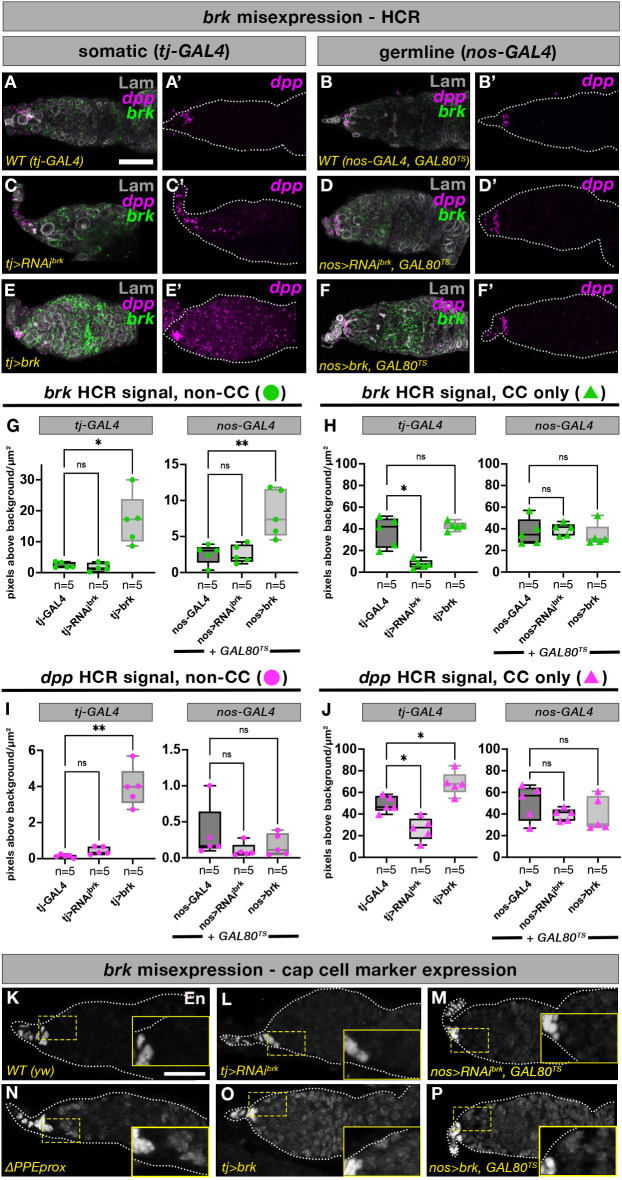
**Upregulation of *brk* in the somatic cells leads to increased *dpp* expression and ectopic expression of the TF/CC marker En.** (A-F′) HCR against *dpp* and *brk* in wild-type *tj-GAL4* or *nos-GAL4* germaria (A-B′), as well as after RNAi against *brk* driven by *tj-GAL4* (C,C′) or *nos-GAL4* (D,D′) or *brk* overexpression driven by *tj-GAL4* (E,E′) or *nos-GAL4* (F,F′). (G-J) Quantification of HCR levels in whole germaria excluding CCs, as well as in CCs alone, was performed for both *brk* (G,H) and *dpp* (I,J). (K-P) Staining for the CC marker Engrailed (En) in wild-type (K), *tj>RNAi^brk^* (L), *nos>RNAi^brk^, Gal80^TS^* (M), Δ*PPEprox* (N), *tj>brk* (O) and *nos>brk, Gal80^TS^* (P) germaria. Insets show magnified views of the boxed regions. One-way ANOVA was used for statistical comparison of each dataset to its respective control genotype (see Materials and Methods). *n*=number of germaria; box plots extend from the 25th to 75th percentiles with the horizontal line indicating the median and with whiskers indicating minimum and maximum values (see Table S2). Scale bars: 20 μm.

The Δ*PPEprox* mutant supports increased levels of *brk* in both the soma and the germline, leaving open the possibility that the increased expression of *dpp* observed in this mutant is non-cell-autonomous. Therefore, we again performed *brk* overexpression and RNAi experiments using both *tj-GAL4* and *nos-GAL4* (constrained to the adult using *GAL80^TS^*) and performed HCR analysis to assess the effects on *dpp* expression. In concordance with the increased pMad^+^ cell number we observed upon *tj*-driven *brk* overexpression (Fig. 5D,E), we also observed a corresponding increase in expression of *dpp* in the ECs of these germaria (Fig. 6E,E′, compared with A,A′, I), supporting the conclusion that increased *brk* upregulates *dpp* in the same cells (Fig. 6G-J). Notably, because *tj-GAL4* is only expressed at low levels in the CC, we did not observe a significant increase in *brk* in the CCs upon *tj*-driven *brk* overexpression (Fig. 6E,H), yet there was still a significant increase in *dpp* expression in these cells (Fig. 6E′,J). Similarly, the loss of *dpp* in the CCs in the *tj>brkRNAi* (Fig. 6C,C′ compared with A,A′, J) is consistent with the loss of pMad^+^ cells in this genotype (Fig. 5C,E). As discussed in the preceding section, this change is likely at least partially due to an earlier role for *brk* in the pupal ovary.

To investigate further the mechanism for the pMad^+^ cell increase in the Δ*PPEprox* mutant, we used RNAi to knock down *dpp* in the mutant background with two different FC drivers (*tj-GAL4* and *GMR2A11-GAL4*). In both cases, the number of pMad^+^ cells in the Δ*PPEprox* was decreased, mirroring the effect of the *dpp* RNAi in the wild-type background, indicating that tumorous germaria in the Δ*PPEprox* mutant are dependent on the levels of *dpp* in the ECs (Fig. S5). In contrast, neither upregulation nor knockdown of *brk* in the germline changed the expression of *dpp* in either the ECs or the CCs (Fig. 6B,B′,D,D′,F-J), indicating that the increase in pMad^+^ cells observed in *nos>brkRNAi* germaria (Fig. 5H,J) is independent of *dpp* levels.

### Increased *brk* expression in either the germline or the soma may alter EC fate and effectively expand the GSC niche

To gain insight into the specific function of Brk in the germline, we again generated germline *brk* mutants, as well as performing RNAi and overexpression using both somatic and germline drivers, and examined readouts of key signaling pathways and established markers of germarium morphology. We found that increased *brk* expression, either ubiquitously (Δ*PPEprox*) or specifically in either the somatic cells (*tj>brk* and *C587>brk, Gal80^TS^*) or the germline (*nos>brk, GAL80^TS^*), leads to posterior expansion of the CC marker Engrailed (En) (Fig. 6N-P compared with 6K), with no such effect apparent upon RNAi (Fig. 6L,M). Additionally, we observed enrichment of the *Drosophila* E-Cadherin Shotgun (Shg), in ECs in all of the conditions in which *brk* is upregulated in the EC (i.e. Δ*PPEprox* mutants, *tj>brk* and *C587>brk, Gal80^TS^*) compared with wild type (Fig. S6A-D′). Shg is a key component of adherens junctions, which are important in wild-type germaria to maintain GSC contact with cap cells so that they remain within range of the *dpp* self-renewal signal (Song et al., 2002). These results suggest that precise regulation of *brk* levels in the somatic cells of the germarium is important for EC fate (see Discussion).

**Fig. 7.**
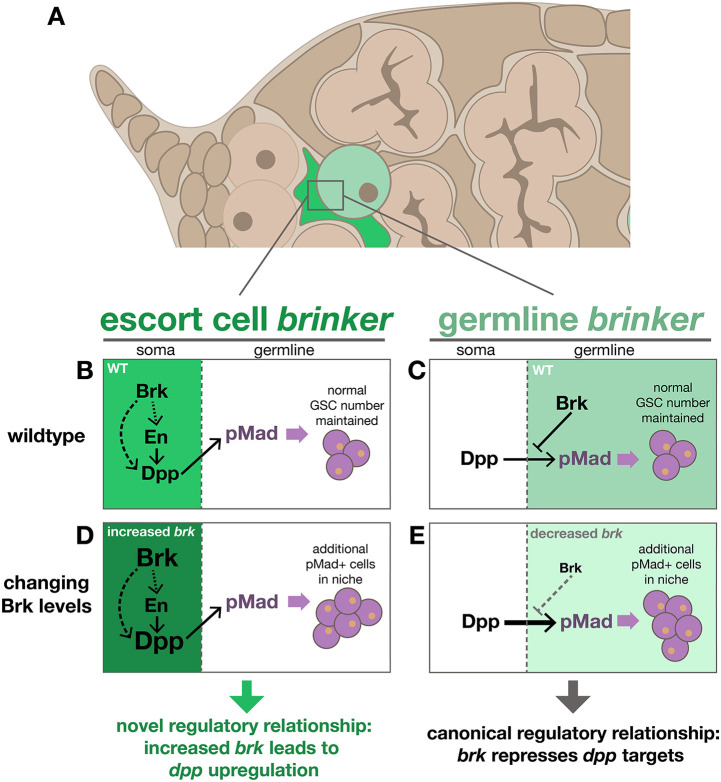
**A model for the tissue-specific role of Brk in germline homeostasis.** (A) Schematic of the cell types in the anterior germarium with emphasis on ECs (green) and pMad^+^ cells (light green) that express *brk*. (B-E) Regulatory diagrams of the role of Brk in regulation of BMP signaling to affect pMad^+^ cell number in wild-type somatic cells (B), in wild-type germline cells (C), in somatic cells (e.g. ECs) when Brk levels are increased (D), and in germline cells (i.e. pMad^+^ cells) when Brk levels are reduced (E). This role in the soma (B,D) represents a previously unknown regulatory role for Brk.

Consideration of tissue-specific perturbation of *brk* levels and the resulting effect on germ cell number (Fig. 5) as well as on *dpp* levels (Fig. 6) sheds light on the seemingly contradictory observation that all constitutive PPE mutants have increased numbers of pMad^+^ cells (Fig. 3) despite having opposing effects on *brk* levels (Fig. 4F). The increase in pMad^+^ cells observed in the Δ*PPE2kb* and Δ*PPEdist* mutants can be explained by loss of germline Brk, resulting in de-repression of BMP signaling. This is supported by our germline-specific *brk* misexpression experiments (Fig. 5J; Fig. S3E) and represents the expected result for Brk acting in its canonical role as a repressor of *dpp* and its targets (e.g. Fig. 7C,E). Conversely, increased pMad^+^ cell numbers observed in Δ*PPEprox* mutants is caused by increased EC *brk* expression, which we show results in increased *dpp* (Fig. 4E,G). This phenomenon is recapitulated by our somatic *brk* misexpression experiments (Figs 5E; 6E,I,J; Fig. S3A,B) and represents a previously unappreciated regulatory relationship between *brk* and *dpp* (e.g. Fig. 7B,D).

## DISCUSSION

*brk* expression in the ovary depends on a cis-regulatory PPE, which has pleiotropic and multimodal functions. Previously identified in the early embryo as required for the action of two distal enhancers (Dunipace et al., 2013), here we show that the PPE also performs that function in the ovary where it supports enhancers active in CFCs. In the germarium, however, the PPE also itself acts as a driver of expression, supporting expression of *brk* within the soma as well as the germline. Furthermore, the proximal and distal PPE domains interact to finely tune levels of *brk* output. This regulatory role is crucial as we find that perturbation of *brk* levels in either direction – in soma or germline – significantly impacts germ cell homeostasis. Somatic *brk* levels also have important implications for follicle cell development in later-stage egg chambers and for fertility. Analysis of *brk PPE* mutants as well as cell type- and developmental stage-specific perturbation of *brk* levels demonstrate that Brk is a near-ubiquitous factor in the ovary with broad impacts on development.

In other developmental contexts, Brk has been shown to act primarily as a repressor of the BMP ligand Dpp and its targets, often to shape morphogen gradients formed as a result of long-range Dpp signaling. We were therefore not surprised to find that *brk* is expressed in the ovary, where *dpp* is known to play an important role as a short-range signal that supports GSC maintenance. What was unexpected, however, was the extent to which *brk* and *dpp* expression domains overlap. Our results establish that Brk acts as a positive regulator of *dpp* in the germarium and itself has an important role in regulating germline homeostasis. This role reversal of Brk, from its canonical function as a repressor of *dpp* and its targets, to a positive regulator (Fig. 7A,B,D) appears to be confined to the germarium; in other ovary tissues where BMP signaling is important (e.g. egg chambers stage seven and older), *dpp* and *brk* domains are mutually exclusive (Fig. S2A-B″).

This unexpected *brk* and *dpp* co-expression in wild-type germaria, as well as our finding of *dpp* upregulation in response to *brk* overexpression, aligns with the phenotypes we observed in mutants that lead to somatic *brk* overexpression (i.e. Δ*PPEprox* and *tj>brk*), including increased GSC number. Taken together, these observations support a model in which somatically expressed Brk promotes escort cell *dpp* expression (Fig. 7B,D), which effectively expands the niche by providing self-renewal signals throughout the anterior germarium. This model is further supported by the expansion of the cap cell marker En into the escort cell domain in *brk* overexpression mutants (Fig. 6N,O). En has also been demonstrated to be necessary and sufficient for the expression of *dpp* in the germarium (Luo et al., 2017), indicating that the upregulation of *dpp* as a result of *brk* overexpression could also be an indirect effect mediated by En (Fig. 7B,D).

Enrichment of Cadherin in the escort cells in Δ*PPEprox* mutants, which overexpress *brk*, suggests that these cells are not only changing their gene expression programs as a result of higher *brk* but are also adopting morphological properties key to the cap cells' biophysical function in the niche (Fig. S6B). These abnormal escort cells present in Δ*PPEprox* mutants, which display mixed characteristics of both CCs and ECs, are similar to the phenotype observed in mutants for the Swi/Snf chromatin remodeling complex protein Osa, which exhibited a similar expansion of En and *dpp* expression into ECs, as well as an increased number of undifferentiated germ cells, yet continued to express EC markers such as the *c587-GAL4* reporter (Hu et al., 2021). Epigenetic modifications in general are crucial for germ cell maintenance, and expansion of BMP signaling in the niche is a common phenotype of misregulation of these factors (e.g. Eliazer et al., 2014; Wang et al., 2011; Yang et al., 2017). These broad changes in gene expression suggest that Brk is required for EC differentiation and/or chromatin remodeling.

Our observation of increased pMad^+^ cell number in germline-specific *brk* mutants or RNAi was surprising given our findings that increased *brk* expression in the soma is correlated with increased pMad^+^ cell number, indicating that Brk has opposite effects in these closely connected tissues. This finding, however, aligns with our analysis of the constitutive PPE mutants in which deletion of the full-length, distal or proximal PPE domains all resulted in increases in pMad^+^ cell number, even though these deletions had opposing effects on *brk* expression levels. Taken together, these results indicate that proper regulation of *brk* in both the germline and soma is collectively important and that relative expression levels may be more important than the absolute expression in a single tissue. Brk and Dpp are known to be involved in cell-cell competition and survival in other tissues in which the relative expression level from the neighboring cell is important in determining the fate of a given cell (Moreno et al., 2002). Additionally, in the testis, ectopic Dpp signaling in cyst stem cells (CySCs) resulted in CySC-GSC competition and GSC loss (Lu et al., 2019), indicating that the signaling between the germline and soma can result in cell-cell competition in the opposing tissue. Based on our findings in this study, it is probable that the relative expression levels of *brk* between the germline and somatic cells is involved in stem cell competition and important for GSC homeostasis. Furthermore, if, as in the wing disc, the upregulation of *brk* is required for apoptosis of less-fit cells in a tumorigenic tissue (Moreno et al., 2002) then loss of *brk* in the germline could indeed lead to excess GSCs.

## MATERIALS AND METHODS

### Fly stocks and genetics

All flies used were strains of *Drosophila melanogaster* and reared at 25°C on standard fly media. See Table S1 for details regarding stocks used.

### Transgenic reporter lines

Large reporter constructs have been described by Dunipace et al. (2013). The H2A-mCherry small reporter construct was made by subcloning the *hsp70* promoter from pUASBP (Horne-Badovinac and Bilder, 2008) into the attB vector (Bischof et al., 2007), and subsequently cloning the H2A-mCherry reporter with associated SV40 terminator (Kadam et al., 2012) downstream of the promoter. PPE small reporter fragments were amplified by PCR from *brk-gfp* (Dunipace, et al., 2013) and cloned into the H2A-mCherry vector. All reporter constructs were injected into y^1^ M{vas-int.Dm}ZH-2A w*; M{3xP3-RFP.attP}ZH-86Fb flies [Bloomington *Drosophila* Stock Center (BDSC) #24749].

### Genomic CRISPR deletions

For deletions within the genome, gRNA constructs were created by modifying the pCFD4 plasmid (Addgene plasmid #49411) to target PAM sequences flanking the region to be deleted. flyCRISPR Optimal Target Finder (https://flycrispr.org/target-finder/) was used to identify the PAM sequences with no predicted off-target hits. The gRNA plasmids were then injected into *y2 cho2 v1 P{nos-phiC31\int.NLS}X; attP2 (III)* (NIG-Fly #TBX-0003) flies. Stable gRNA transgenic lines were created and then crossed to a Cas9-expressing line (*y2 cho2 v1; Sp/CyO, P{nos-Cas9, y+, v+}2A*, NIG-Fly #Cas-0004). Individuals from the next generation were screened by PCR for the deletions (see Table S3 for primers and genomic sequence of mutants).

### RNAi and misexpression

Flies containing either P{UAS-brk.3PF3} (*brkUAS.Tag:HA*, BDSC #78350) or P{NIG.9653R} (UASt-dsRNA against *brk*, NIG-FLY #9653R-2) transgenes were crossed to flies containing the desired GAL4 driver (*tj*-, *c587*-, *GMR25A11*-, *bab1*-, *109-30*-, *nos*- or *bam*-*GAL4*). F1 females of the appropriate genotype were collected and raised at 27°C on standard fly media supplemented with yeast paste together with males to promote robust egg production. For genotypes indicated in Figs 5, 6, S3 and S4, *GAL80^TS^* was used to limit activity to the adult ovary by crossing flies at 18°C and then shifting to 29°C after eclosion. After 3-5 days, ovaries were dissected from these females as described below. Each *GAL4* line alone or *GAL4, GAL80^TS^* line (with parallel temperature shifts) was stained and used for control pMad^+^ cell counts (Fig. 5 and Fig. S3, ‘GAL4 controls’).

### Generation of mitotic clones

To generate cell type-specific mitotic clones of *brk* Δ*PPEprox*, we recombined CRISPR-Cas9 deletions with FRT18A (BDSC #5245). We then crossed this recombinant, as well as previously made *brk-*FRT recombinants (see Table S1), to a UAS-FLP fly strain (BDSC #4539 or 4540) to generate stocks of the generic genotypes brk* (either gene null mutant or *PPEprox* deletion), *FRT18A; UAS-FLP* and y*, w*, ubi-GFP (BDSC #5245 or #5624), *FRT18A; X-GAL4* (where *X* indicates either *tj*, *nos* or *109-30*). Appropriate combinations were crossed to obtain germaria mosaic for *brk* in GAL4-expressing tissues. Although two different GFP markers were used to visualize clones, neither was strongly expressed enough to observe mosaicism consistently in germaria. We confirmed that our system was working to generate mosaics by observing later-stage egg chambers where the GFP marker was clearly visible and clones could be observed, indicating that recombination was occurring at earlier stages. For *brk* mutant clones, one of two null alleles were used (*brk^XA^* or *brk^KO^*), except in the case of *nos-GAL4* experiments for which data from both mutants was pooled, i.e. ‘*brk^−^*’, as cell count distributions did not differ between mutants. Control crosses were performed using identical genotypes but lacking UAS-FLP (Fig. 5 and Fig. S3, ‘FRT controls’).

### Immunostaining and HCR

Ovaries were collected from 3- to 5-day-old flies aged at 25°C on standard food supplemented with yeast. Ovaries were dissected in cold EBR (0.13 M NaCl, 4.7 mM KCl, 1.9 mM CaCl_2_, 10 mM HEPES, pH 6.9) and then fixed for 20 min at room temperature in PBS with 0.1% Tween 80 (PBT), 4% paraformaldehyde and 1% DMSO. Tissues were washed three times, 5 min each, in PBT and then incubated for 1 h in 1% Triton X-100 in PBS. Three more five-minute washes with PBT were followed by incubation for 1 h in 1× Western Blocking Solution (Millipore/Sigma, 11921673001) in PBT (WB). The tissues were incubated overnight at 4°C with primary antibody in WB. The following day the samples were washed three times, 10 min each, and then blocked in WB for 30 min. The tissues were incubated with secondary antibodies overnight at 4°C in WB. Finally, the tissues were washed three times, 10 min each, with PBT, incubated with DAPI in PBT for 30 min, washed a final two times, 5 min each, with PBT and mounted in 70% glycerol/30% PBS. HCR was carried out as described by Slaidina et al. (2020) using *brk* 4049/E190 and *dpp* 4049/E192 probes from Molecular Technologies, and mounted in Slow Fade Gold (Thermo Fisher, S36936).

Primary antibodies used in this study were: α-Spectrin (1:100, 3A9), Lamin C (1:50, LC28.26), Lamin (1:100, ADL84.12), Bam (1:10), En (1:20, 4D9), E-Cadherin (1:50, DCAD2) and Vasa (1:50) from the Developmental Studies Hybridoma Bank; pSMAD1/5 (1:50, Cell Signaling Technology, 9516), RFP (1:1000, MBL International, PM005), GFP (1:5000, Rockland Immunochemicals, 600-101-215) and Tj (1:5000, kindly provided by the Godt Lab; Li et al., 2003). Secondary antibodies (1:400) were conjugated to Alexa Fluor 488, Alexa Fluor 555 or Alexa Fluor 647 (Molecular Probes, Life Technologies) and used with DAPI (Invitrogen, D3571). All antibodies used have been previously published and were validated by confirming expected expression patterns in wild-type tissues.

### Confocal microscopy and image processing

All images were captured using a Zeiss LSM 800 confocal microscope and associated Zeiss microscope software (ZEN blue). All images, except for HCR experiments, were captured using a 20× objective, N.A 0.75. HCR images were captured with 63 ×oil objective, N.A. 1.4, using a scan speed of 7 and 8 times averaging.

### Quantification and statistics

All female flies of the appropriate genotype that eclosed within a 2-day window were collected, dissected and stained. In all cases this was at least three individuals (i.e. biological replicates). From these collections, all unobstructed germaria in which relevant structures were visible on the slide were imaged and quantified. Power analysis performed using the G*Power tool (version 3.1; Faul et al., 2007) on preliminary data collected for pMad^+^ cell number in PPE mutants suggested that a sample size of ∼11 samples per group would be sufficient to detect the differences in means (effect size ∼0.8) with high statistical confidence (1−β err prob=0.95). As more subtle effects were likely with misexpression and mosaic tissue-specific experiments, we more than doubled this *n* for all experiments in which pMad^+^ cell numbers were counted. Similar analyses indicated that our sample sizes were more than sufficient for other phenotypes quantified: H2A intensity and egg chamber number. For HCR analysis, acquisition of sufficiently high-resolution scans of ovarioles is time consuming and therefore this constraint determined sample size for these experiments. Still, we detected statistically significant differences between samples that appeared visually different, indicating that our sample sizes were sufficient to capture real differences in HCR signal. Operators were not blinded to sample genotype.

PPE H2A-mCherry reporter expression was quantified using Imaris software using the spot detection tool to find all spots with an estimated 3 μm diameter. The mean intensity for each spot was determined and the cap cells were manually defined using LamC staining. Undifferentiated germ cell counts were obtained from maximum projections of confocal stacks of germaria stained for pMad, as well as Spectrin and Lamin C to mark the niche. HCR signal for *dpp* and *brk* probes was quantified using Zen software. A maximum intensity projection of 16 *z*-stacks (0.5 µm each) was made and a region of the germarium encompassing regions 1-2a, but excluding the CCs, was drawn and measured using the spline contour tool. All pixels of a given wavelength with a signal intensity above a threshold of 30 were counted and the total count was divided by the area to give the final measurement. The threshold was chosen empirically by examining ten images from different genetic backgrounds and choosing the lowest intensity at which a spot could be detected by eye. This process was repeated for the CC analysis.

All statistical tests and data visualizations were performed using Prism software (version 9.1.0, www.graphpad.com). Reporter intensity, GSC number and egg chamber number were compared between genotypes or experimental conditions using ordinary one-way ANOVA, and HCR signal with Brown–Forsythe ANOVA, whereby each experimental condition was compared with the matched control (see Table S2). *P*-values were corrected for multiple comparisons using Dunnett's test. HCR quantification for *dpp* in the non-CC domain of Δ*PPEprox* germaria was additionally compared with wild type using an unpaired, two-tailed *t*-test. These data show a clear increase in *dpp* signal intensity compared with wild type that was not captured as statistically significant with our stringent *P*-value correction approach using ANOVA (see Fig. 4G ‘ns’). We provide the significant *P*-value resulting from this *t*-test in Table S2 to support our assertions in the text that the Δ*PPEprox* mutant has elevated *dpp*. Statistical significance of *P*-values (GraphPad format) is abbreviated on plots as follows: not significant (ns) where *P*≥0.05, significant (*) where 0.01≤*P*<0.05, very significant (**) where 0.001≤*P*<0.01, extremely significant (*** and ****) where 0.0001≤*P*<0.001 and *P*<0.0001, respectively. Error bars represent mean ±s.d. Box plots extend from the 25th to 75th percentiles with the horizontal line indicating the median and with whiskers indicating minimum and maximum values (Figs 4 and 6). See Table S2 for *n* (number of nuclei, ovarioles or germaria, as appropriate) and corrected *P*-values for all genotypes and conditions analyzed.

## Supplementary Material

Supplementary information

Reviewer comments
