# Supplementary material for: brinker levels regulated by a promoter proximal element support germ cell homeostasis
Source: Development. 2022 Feb 4;149(3):dev199890. doi: 10.1242/dev.199890 (PMC8918798; doi:10.1242/dev.199890)
Supplement: Supplementary information [file develop-149-199890-s1.pdf]

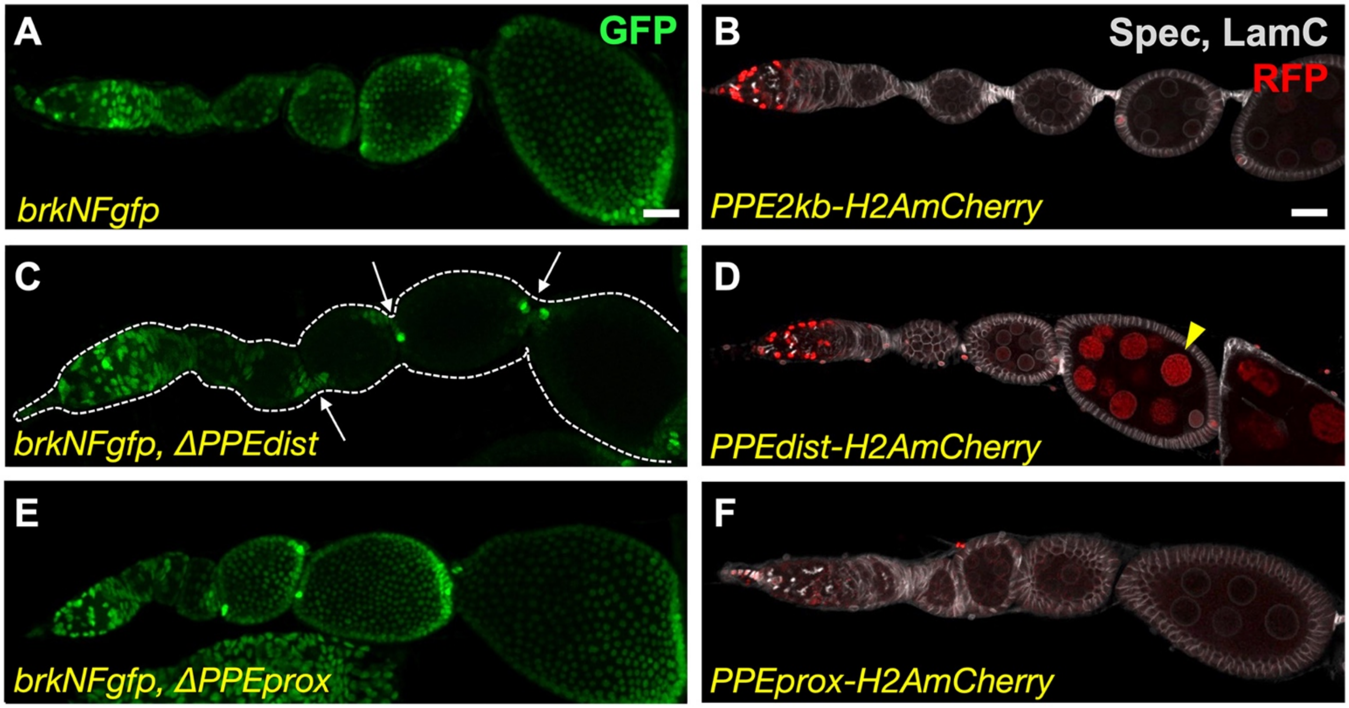

Fig. S1. **PPE and *brk* reporter expression in mid-stage egg chambers.** Large *brk* reporter expression in mid-stage egg chambers from wildtype *brkNFgfp* (A) or when *PPEdist* (B) or *PPEprox* (C) is deleted. White arrows indicate polar cell (PC)/border cell (BC) expression that persists in *brkNFgfp-ΔPPEdist*. Direct fusion reporter expression for full length (2kb) *PPE* (D), *PPEdist* (E) and *PPEprox* (F) in mid-stage egg chambers. Yellow arrowhead indicates germline expression that is associated with *PPEdist-mCherry* only. In all images, anterior is left and scale bar = 20 µm.

Development • Supplementary information

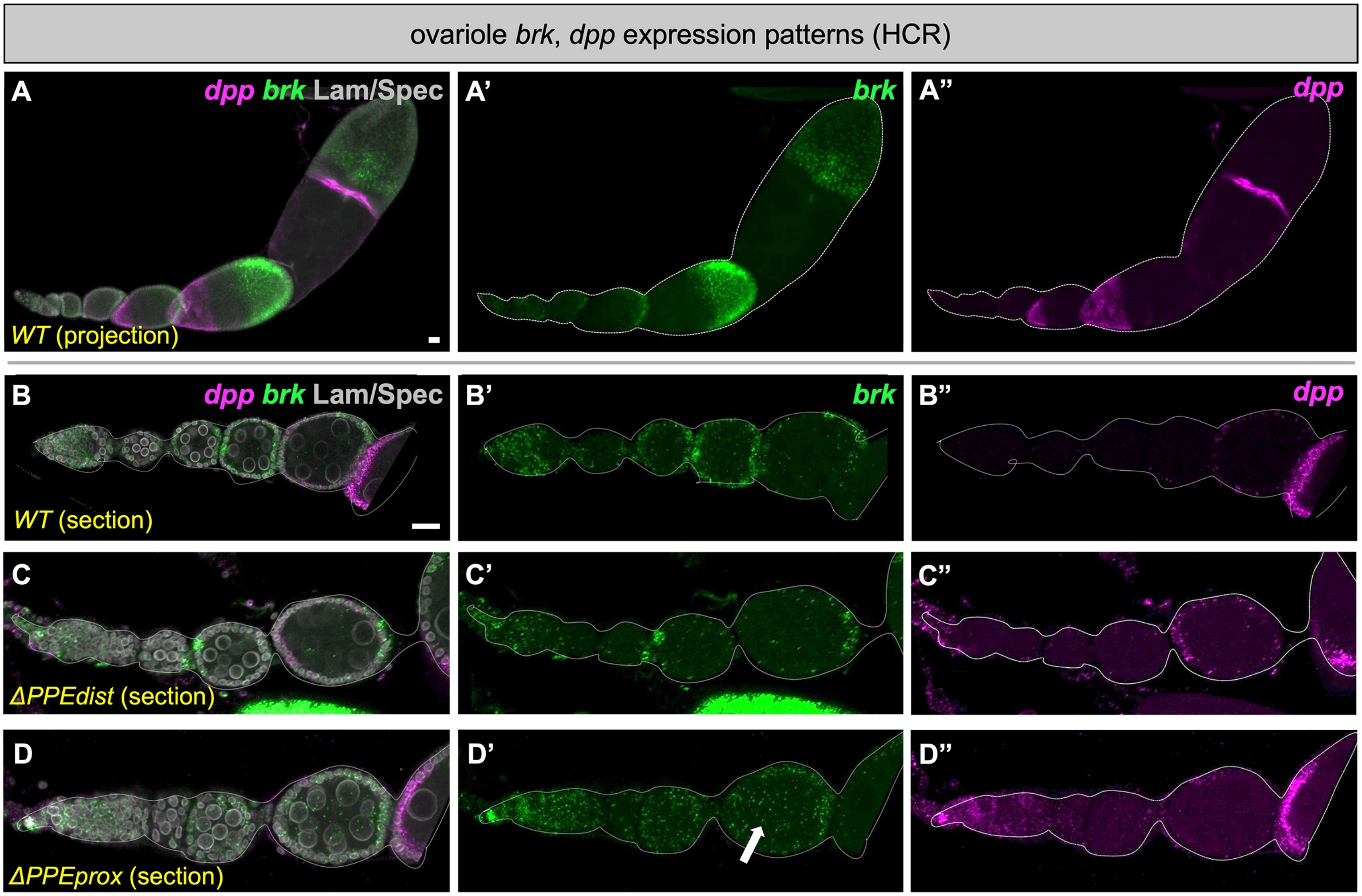

Fig. S2. *dpp* and *brk* are not co-expressed in mid- to late-stage egg chambers. Expression pattern of *brk* and *dpp* as visualized by HCR and co-stained with Lam, Spec in WT (*yw*: maximum projections, A-A” and sections B-B”), *ΔPPEdist* (C-C”) and *ΔPPEprox* (D-D”) shows that *dpp* and *brk* domains do not overlap in mid-late stage egg chambers. Germline *brk* expression indicated with white arrow and is only associated with *ΔPPEprox* mutant background. Scale bar = 20 µm.

Development • Supplementary information

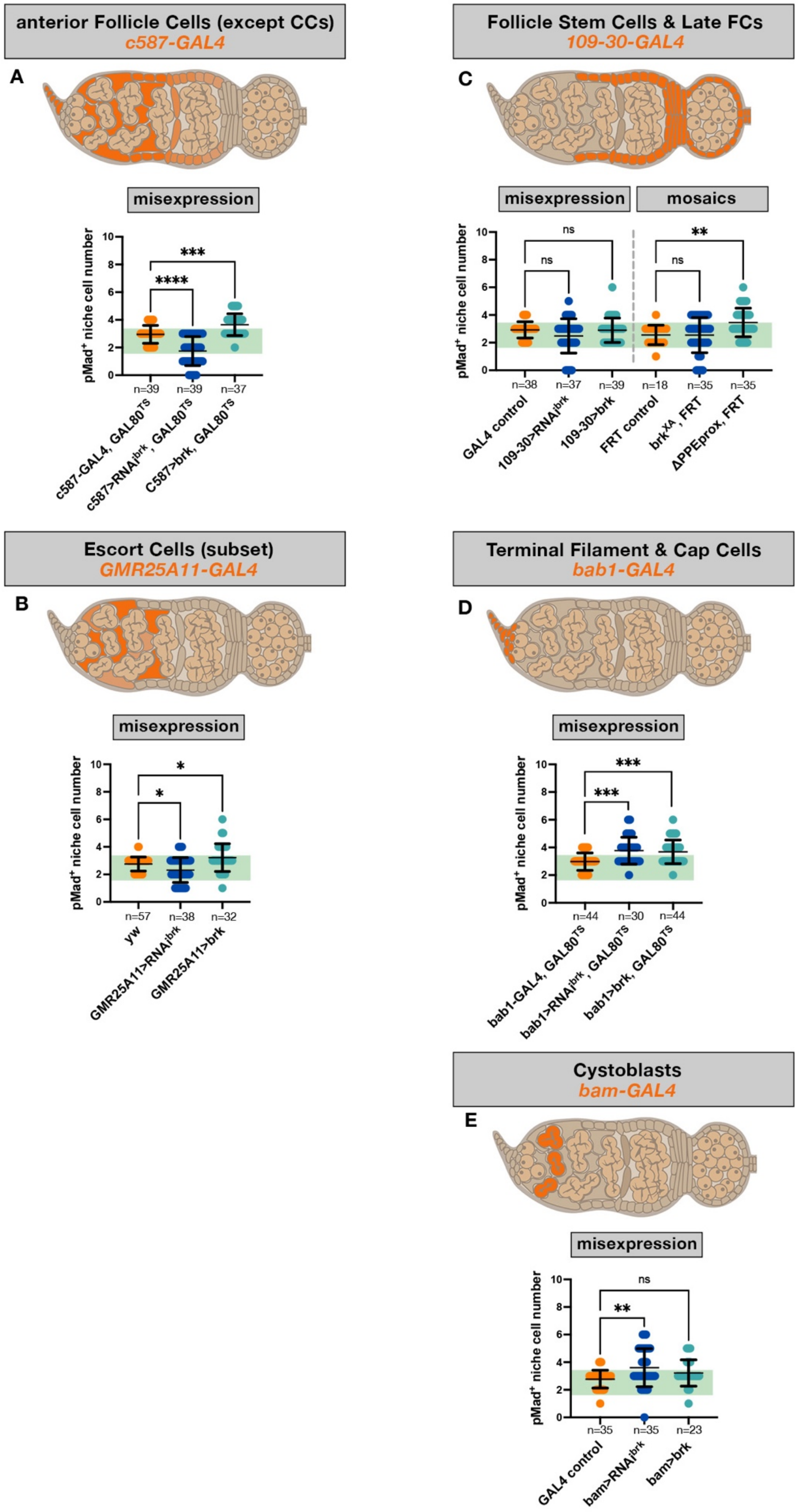

Development • Supplementary information

Fig. S3. Tissue-specific perturbation of *brk* expression with additional drivers supports a role for *brk* in the anterior ECs and the germline for maintaining germ cell homeostasis. Schematics showing expression patterns of additional GAL4 drivers used to affect *brk* expression in somatic and germline sub-populations and the resulting quantification of $pMad^+$ niche cells: (A) anterior germarium follicle cells, except cap cells (*c587-GAL4*), (B) a subset of region 1 and 2a escort cells (*GMR25A11-GAL4*), (C) follicle stem cells and their progeny (*109-30-GAL4*), (D) terminal filament and cap cells (*bab1-GAL4*), and (E) the cystoblast region of the germline (*bam-GAL4*). The number of pMad+ cells in the niche region was quantified for each genotype and plotted; green bar signifies "normal" range of 2-3 pMad+ cells per germarium. One-way ANOVA was used for statistical comparison of each dataset to its respective control genotype (see Methods). n = number of germaria, error bars represent mean±s.d. (see Table S2).

Development • Supplementary information

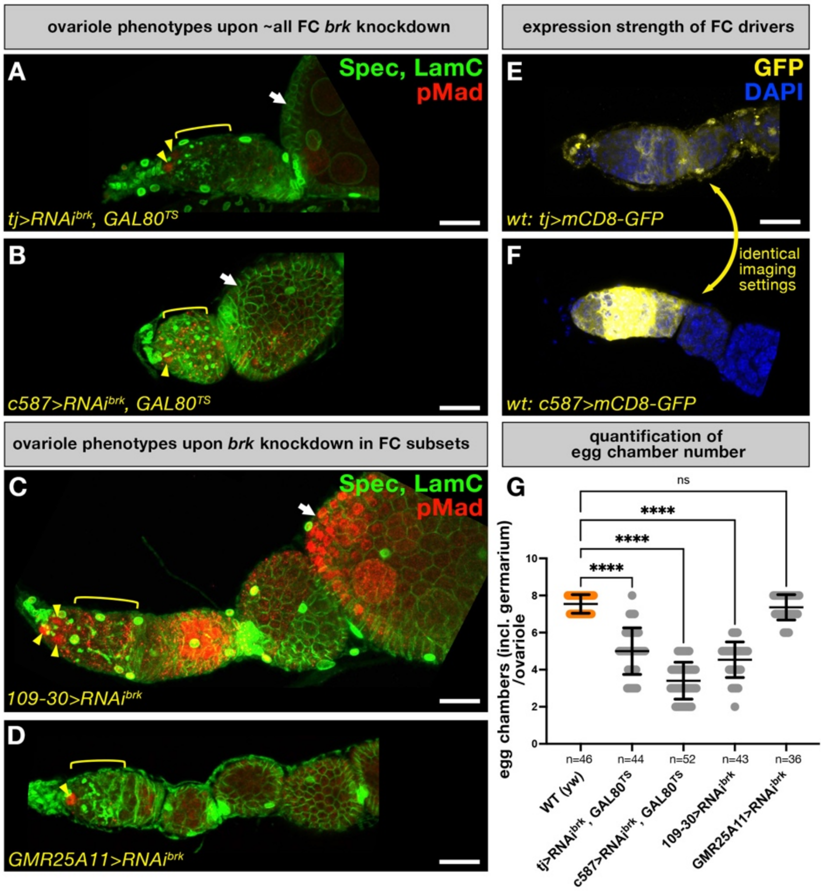

Fig. S4. Loss of *brk* in follicle cells leads to mid-late stage follicle phenotypes that are separable from pMad+ cell regulatory role. Representative germaria resulting from broad FC *brk* knockdown driven by either *tj-GAL4* (constrained to adult using *GAL80*^{TS}; A) or *c587-GAL4* (constrained to adult using *GAL80*^{TS}, B) have different effects on pMad+ cell number (as visualized by pMad+ staining in the niche; indicated by yellow arrowheads - see also Fig. S3); while both lead to loss of mid-stage egg chambers (late stage egg chambers abutting germaria indicated with white arrows) as well as perturbed or absent fusomes (indicated by yellow bracket). These drivers differ in strength, as indicated by signal from UAS-mCD8-GFP (E, F), in a trend that corresponds to the severity of their effects on pMad+ cell number. Drivers active in subsets of follicle cells: *109-30-GAL4* is expressed in FSCs and shows a loss of mid-stage egg chambers similar to that of *tj-GAL4-* and *c587-GAL4*-driven *brk* RNAi (C). Whereas *GMR25A11* which is active in anterior ECs has a moderate effect on pMad+ cell number but mid to late-stage egg chambers appear normal (D). In all images, anterior is to the left and scale bars = 20μm. (G) Number of egg chambers (including the germarium) per ovariole was quantified for follicle cell *brk* knockdown and one-way ANOVA was used for statistical comparison of each dataset to the control genotype (*yw*; see Methods). n = number of ovarioles, error bars represent mean±s.d. (see Table S2).

Development • Supplementary information

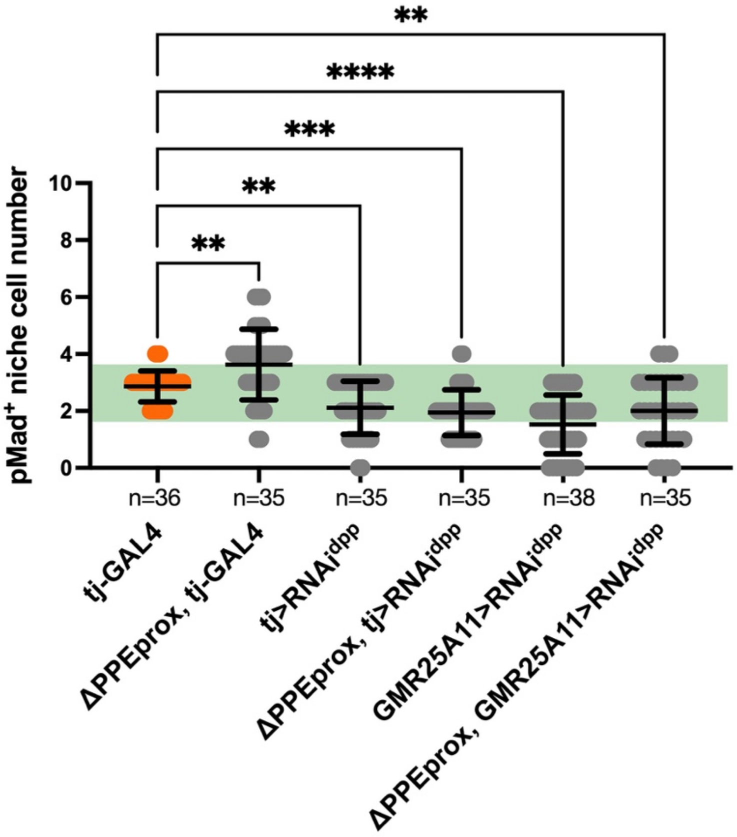

Fig. S5. *dpp* RNAi demonstrates that increase in pMad+ cell number associated with *ΔPPEprox* mutant relates to *dpp*. RNAi against *dpp* was performed using either *tj-GAL4* or *GMR25A11-GAL4* in wildtype and *ΔPPEprox* mutants (without *GAL80*^{TS}). *ΔPPEprox, tj-GAL4* control has significantly more pMad+ cells than wildtype but *dpp* RNAi with either *tj-GAL4* or *GMR25A11-GAL4* in the mutant background mirror the knockdown in a wildtype background, indicating a dominant effect of *dpp* in the EC. One-way ANOVA was used for statistical comparison of each dataset to control genotype (*tj-GAL4*; see Methods). n = number of germaria, error bars represent mean±s.d. (see Table S2).

Development • Supplementary information

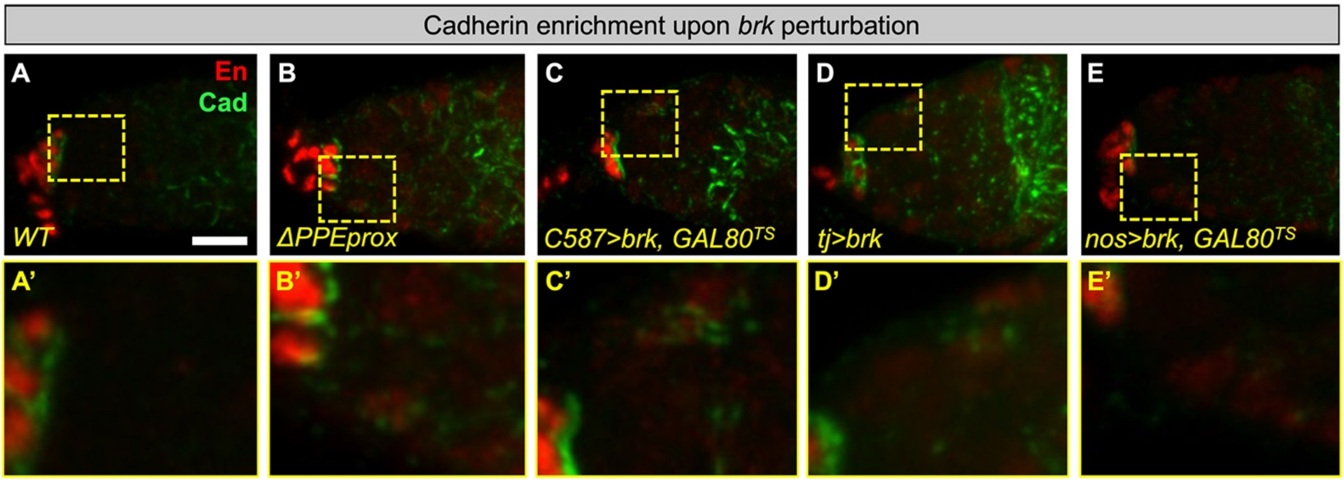

Fig. S6. Expression of additional cap cell markers in ECs suggest these cells have altered fate when *brk* is upregulated. Staining for cap cell markers En and Cad in wt (A,A') compared to mutant conditions shows enrichment of Cad at ECs where En is upregulated (see also, Fig. 6K-P) in *ΔPPEprox* (B,B'), and upon *brk* overexpression driven by either *c587-GAL4* (constrained to adult with *GAL80^{TS}*; C,C') or *tj-GAL4* (D,D'), but not when driven by *nos-GAL4* (E,E') . Inset area indicated with yellow dashed box. In all images anterior is left, scale bars = 20 µm.

Table S1. *Drosophila* fly stocks

Click here to download Table S1

Table S2. Detailed statistics

Click here to download Table S2

Table S3. Primers and genomic sequences

Click here to download Table S3

Development • Supplementary information
