# Supplementary material for: brinker levels regulated by a promoter proximal element support germ cell homeostasis
Source: Development. 2022 Feb 4;149(3):dev199890. doi: 10.1242/dev.199890 (PMC8918798; doi:10.1242/dev.199890)
Supplement: Reviewer comments [file develop-149-199890_review_history.pdf]

brinker levels regulated by a promoter proximal element support germ cell homeostasis

Leslie Dunipace, Susan Newcomb and Angelike Stathopoulos
DOI: 10.1242/dev.199890

Editor: Cassandra Extavour

Review timeline
Original submission: 10 June 2021
Editorial decision: 29 July 2021
First revision received: 12 November 2021
Editorial decision: 16 December 2021
Second revision received: 21 December 2021
Accepted: 22 December 2021

Original submission

First decision letter

MS ID#: DEVELOP/2021/199890

MS TITLE: brinker levels regulated by a promoter proximal element support germline stem cell homeostasis

AUTHORS: Leslie Dunipace, Susan Newcomb, and Angelike Stathopoulos

I have now received all the referees' reports on the above manuscript, and have reached a decision. The referees' comments are appended below, or you can access them online: please go to BenchPress and click on the 'Manuscripts with Decisions' queue in the Author Area.

As you will see, the referees express considerable interest in your work, but have some criticisms and recommend revision of your manuscript before we can consider publication. I agree that considering these suggestions would strengthen this interesting MS. If you are able to revise the manuscript along the lines suggested, which may involve further experiments, I will be happy receive a revised version of the manuscript. Your revised paper will be re-reviewed by one or more of the original referees, and acceptance of your manuscript will depend on your addressing satisfactorily the reviewers' major concerns. Please also note that Development will normally permit only one round of major revision.

We are aware that you may be experiencing disruption to the normal running of your lab that make experimental revisions challenging. If it would be helpful, we encourage you to contact us to discuss your revision in greater detail. Please send us a point-by-point response indicating where you are able to address concerns raised (either experimentally or by changes to the text) and where you will not be able to do so within the normal timeframe of a revision. We will then provide further guidance. Please also note that we are happy to extend revision timeframes as necessary.

Please attend to all of the reviewers' comments and ensure that you clearly highlight all changes made in the revised manuscript. Please avoid using 'Tracked changes' in Word files as these are lost in PDF conversion. I should be grateful if you would also provide a point-by-point response detailing how you have dealt with the points raised by the reviewers in the 'Response to Reviewers' box. If you do not agree with any of their criticisms or suggestions please explain clearly why this is so.

© 2022. Published by The Company of Biologists under the terms of the Creative Commons Attribution License (https://creativecommons.org/licenses/by/4.0/).

Reviewer 1

Advance summary and potential significance to field

In this work, the AUs utilize a number of tools available to dissect the role of brk in the ovarian niche. In addition, the AUs generate useful, clean deletions of the PPE element and of its two subunits, PPEdistal and PPEproximal. The most interesting finding of the whole work is the description of a positive regulatory role for brk on dpp expression in the niche, a novel function for this canonical repressor of the dpp pathway. However this novel function is based on in situ hybrydizations that “look” different but that have not been quantified.
This important finding deserves stronger experimental evidence backing it.
I find the MS difficult to read and lacking significant quantifications. In addition, the AUs correlate numbers of GSCs present in the niche with that of pMad+ cells, something that is incorrect. As it stands, I do not find the MS fit for publication.

Comments for the author

General comments:
-The sample size of most of the quantifications is low. Of the 32 genotypes listed in Table S3, only one has an n<20. The MEAN sample size is 13.5+-4.1 (SD). In my opinion, the minimum n to describe accurately a GSC phenotype is 35 germaria, especially considering the variability found in controls. For instance, an increase in sample size is especially important in Fig 2U, that sets the baseline of changes in GSC number for the rest of the paper; and in this case the sample size is between 8 and 11 germaria per genotype. Far too low for the standards in the field. Also, please give the accurate MEAN+-SEM or SD in the text or figure legends, as graphs are not precise enough to know whether a genotype has, e. g., 2.6 or 2.2 GSCs on average.
-Lines 162-164: In general, I am missing quantification of some of the results reported. This is one of such examples: “The distal region of the PPE alone (PPEdist, Fig. 1A) drives higher levels of expression in the CCs than is observed for the full PPE2kb, and also drives EC expression comparable to that of PPE2kb (Fig. 1F, K compared to G)”. Still, this is documented only with 2 examples shown in fig. 1. If the AUs are to claim that deletion of one of the PPE subunits increases or decreases levels of expression, this should be quantified. The same applies to other deletion or reporter constructs (PPEdist>mCh, etc).
-Line 194 and others, fig. 2U: pMad labels GSCs and some cystoblasts (Song et al., Development 2004). To use pMad as a proxy for GSC numbers is incorrect. This applies to several instances in the text, including the final Model and large sections of the DIscussion, in which the AUs talk in terms of GSC numbers. GSCs can only be identified accurately if they possess a spectrosome and are found in contact with cap cells.
-Lines 316-327: The hypothesis of brk acting as a positive regulator of Dpp, while plausible according to the data provided by the authors, is contrary to most of the published literature, so it needs to be backed with more solid evidence. The authors simply rely on qualitative HCR stainings (Figure 4). These need to be quantified, and ideally qPCR quantification of Dpp levels in cap cells should be provided as well. This is particularly important, as this is the most interesting finding of the work.

Specific comments:
-Line 75 and others: Fig 1 calling in the text is confusing. As it stands, the order of citation is 1B, 1C, 1A, 1F, 1G-H, 1D, 1E, 1S-U, 1J, 1V. Please consider rearranging Fig 1 so that panels are cited in the text in an orderly manner.
-Fig. 1B: What does the darker cell next to the GSCs indicate? It is confusing that it has the same colour as the oocyte of later stages. Oocyte in S2 egg chamber should be at the posterior.
-Figs 1D-E, 1G-R are difficult to see as they are very small (and, in this particular pdf file, resolution is pretty poor). Also, I believe the introduction to TF+cap cells and to Escort cells needs panels with separate channels.
For instance, one panel for the tj signal, another for the LamC signal and another one for the brkNFgfp signal.
-Lines76-77: I would not say that a GSC “differentiates” into a CB in physiological conditions. The germarium supports 1) the differentiation of one of the GSC daughters into a CB and 2) the maintenance of the GSC lineage by the other.

© 2022. Published by The Company of Biologists under the terms of the Creative Commons Attribution License (https://creativecommons.org/licenses/by/4.0/).

-Lines 79-81: I would only consider the anterior escort cells as part of the GSC niche, not the rest of the escort cell population, which conform the so-called (albeit I do not like this nomenclature) “differentiation niche”.
-Line 79: Inner, not interior. AlternatiVEly?
-Lines 92-93: Please cite Wilcockson + Ashe, Dev Cell 2019
-Line 99: Consider “cystoblast and cyst development”. There are more relevant papers to refer to the dependence on escort cells of the germline, such as Banisch et al., Dev 2017.
-Line 112: consider “in this tissue”.
-Line 133: “facilatory” typo? What does this sentence mean? Please explain better in the text the difference between a traditional enhancer and a facilitating(?) one.
-Line 138: a-Spec also labels fusomes, not only spectrosomes.
-Lines 140, 141: Please show higher magnifications or larger panels. In their current sizes, it is difficult to distinguish the different cell types.
-Lines:143, 144: No specific expression in polar cells can be observed in the current panels. The S10b shown has GFP staining in all of the columnar follicle cells. Why do the AUs state that “brkNFgfp persists in follicle cells throughout egg chamber development, refining to only the posterior follicle cells (PFC) after stage 7”?
-Lines 126-184: I find the description of the different construct tedious and confusing (I have spent literally hours on these lines, going back and forth between the text, fig 1 and fig S1). I would suggest the AUs the following order: 1) brkNFgfp expression to claim a role for brk in the germarium/ovary, 2) brkNFgfp delta PPE to demonstrate the role of the PPE enhancer, 3) brkNFgfp delta PPE prox and distal to suggest the idea of modality of PPE. Then, use the mCHerry reporters to confirm the previous findings and the brkB data to support multimodality. Of course, this changes in the text should be accompanied by the modification of fig 1.
-Line 187: Which “important developmental process” do the AU refer to? Consider “important structure”.
-Line 189: Please refer to Suppl Material for the exact mapping of the deletions giving rise to the delta PPE2kb, delta PPE dist and delta PPEprox alleles.
-Line 202: The increase in round spectrosomes may indicate as well that there are more GSCs giving rise to more cystoblasts, not necessarily that there is a delay in CB differentiation. In my opinion, it is much more telling the (allegedly) lower numbers of fusomes in the experimental germaria that, if properly quantified could provide evidence to indicate that CB differentiation is truly affected. In addition, in normal conditions GSC differentiation is of little significance.
-Line236: Please quantify.
-Line 258-259: Incorrect. Number of pMad+ cells and number of GSCs are not equal to one another.
-Line 248: Incorrect. Bab1-Gal4 is expressed in escort cells too, albeit with lower levels. In fact, it has been used to generate escort cell clones in addition to TF and cap cell ones.
-Lines 256 and more: It is essential that the AU show that they can generate mosaic germaria. The ought to identify mutant escort cells with the appropriate markers (i. e., lack of GFP or similar to label mutant cells) and correlate loss of brk function with a phenotype in the germ line (if so). As it stands, the experiment in which the AUs look at the consequences for germline development when removing brk from escort cells + cap cells or the germ line is inconclusive.
-How come is Fig S3 cited before Fig S2? The text is difficult to follow, not only because the description of the phenotypes rotates from null conditions to delta PPE to delta PPE dist and delta PPEprox, but also because the order of in text figure calling is chaotic.
-Lines 256 and more: What is the genotype of the flies? How were the clones induced, simply by expressing UAS-FLP under the gal4 drivers, or was tub-Gal80ts also used? This is particularly important in the case of the bab1-Gal4 driver, since TF cells and cap cells do not divide in the adult. And if tub-Gal80ts was not used in conjunction with bab1-Gal4, then the AUs should find escort cell clones when utilizing bab1-gal4. However they report no phenotypes in the germline. This makes even more compelling labelling the clones.
-Line276-277: Are the AUs sure they mean “mislocalisation of the oocyte nucleus”? This is not documented in the figures.
-Line 283 and more: Have the AUs quantified the decrease in brk levels when using the RNAi construct? Or for that matter, the increase in levels after overexpressing UAS-brk? This may help understand the controversy of RNAi and overexpression giving the same gain of GSCs phenotype.
-Line 301: I am not sure I would use the Song et al. reference to cite that Bam is necessary and sufficient for GSC differentiation. Consider works from the McKearin lab.
-Line 338: Incorrect figure calling. Fig 4N does not exist.

© 2022. Published by The Company of Biologists under the terms of the Creative Commons Attribution License (https://creativecommons.org/licenses/by/4.0/).

-Line 339: How do the AUs know that the DE-cad expression shown in the picture at the germline-escort cell boundaries is in the escort cells? It could equally be in the germline. To claim that overexpression of brk induces higher DE-cad levels in escort cells, the AUs should use an escort cell-specific marker in addition to DE-cad. In addition, DE-cad expression is not only important to keep GSCs in their niche, but also to allow the germline cysts to acquire proper morphology, so the argument put forward by the AUs may not be so.
-Line 346, Fig S2N-R: If one is to compare LamC expression levels in two different germaria, cap cell signal should be similar (assuming that the experimental conditions do not affect levels in the cap cells, of course).
And this is not the case in these panels. Please normalize LamC expression with cap cell levels.
-Lines 372-373, Fig. 5B. From the figure, it seems that brk is expressed in hub, CySCs, somatic cells and pre-meiotic 8-16-cell cysts. However, co-stainings with tj and vasa are necessary to confirm the cell types expressing brk. In addition, these co-stainings would help the authors to clarify what they mean with "a subset of somatic cyst cells" (for example, does this mean only early cyst cells?).
-Fig. S5: Vasa staining is of very poor quality. FasIII co-staining with GFP is necessary, as it is difficult to distinguish the position of the hub (if it weren´t for the doted circles, it would be impossible). For example, it is not clear if BrkA is expressed in the hub or in some CySCs.
-Lines 388-391: To address the effects of brk mutants on the male GSCs, the authors simply check pMad levels (Fig S6). There are more direct ways of counting male GSCs (either as Vasa+ in contact with the niche or as round spectrosomes oriented towards the hub) or to check a possible expansion of CySCs (with Zfh1 staining). Also, please double stain the hub, so that the reader knows where to find the GSCs.
-Lines391-394: The AUs claim there is an anterior expansion of Engrailed expression in the testis upon brk overexpression (delta PPEprox) and they draw parallelisms with the ovary. However, according to what is shown in Fig S6E-F, both phenotypes are not comparable. While in the ovary, ectopic Engrailed it is expressed in ECs, in the case of the testis it seems as if it is in germline cells, perhaps in 8-cell transient-amplifying cysts and onwards. In fact, this phenotype does not need to be necessarily explained as an expansion of the domain of En expression: it appears to be simply the result of a reduction in the number of early transient-amplifying cysts. Typically, this could be the result of a reduction in GSC proliferation or numbers, causing late cysts to be closer to the niche.
-Discussion: It is interesting that depending on the Gal4 driver used, brk GOF and LOF show different results.
Thus, while using tj GOF and LOF induces opposite phenotypes in terms of GSC numbers (as expected), both GOF and LOF in the germline (utilizing nos-Gal4) cause an increase in GSCs. The AUs may want to mention this in the Discussion.
-Lines 457-462: some of the mutant phenotypes reported are pleiotropic and indicate "poor cell fitness" of brk mutant cells, raising the question of what are the real effects of eliminating brk in the ovarian niche. This is why labelling the null escort and cap cells in the mosaic germaria is so crucial.
-Fig S2F-G: how do the AUs assay cell death? Please show.
-Fig3E-H lacks images of tj >+ and nos> + controls, necessary for comparison. The same is true for FigS3.
Figure 4 calling in the Discussion is wrong (there are no 4N or 4O panels).

Reviewer 2

Advance summary and potential significance to field

Reveals a role for Brinker in germ cell dynamics in Drosophila ovary Identifies enhancers that drive brk expression in cells in the anterior ovary

Comments for the author

Dunipace et al investigate the role of the transcriptional repressor, Brinker (Brk) in the developing ovary of Drosophila, specifically its role in controlling the ability of germline stem cells (GLCs) to both self-renew and differentiate ultimately into an oocyte and support cells. This is the first report on a role for brk in this system and so it is important that this paper does clearly show that

© 2022. Published by The Company of Biologists under the terms of the Creative Commons Attribution License (https://creativecommons.org/licenses/by/4.0/).

brk is important in this regard. However, as the authors themselves indicate, interpreting the data is difficult and a definitive role(s) for brk in this system remains elusive.
The paper starts off with reporter analysis to show that brk is expressed in cells in the anterior ovary, high in cap cells (CCs), lower in escort cells (ECs) and possibly not in the germline. This analysis is clear and convincing.
Deletion analysis of the relevant enhancer(s) is relevant and good but interpretation is difficult because although these deletions can have opposite effects on brk expression they can have similar cellular phenotypes, i.e. increased number of GLCs, but then have no effect on fertility. As the authors point out, regulation may occur to counteract the earlier affects to allow normal fertility. So this section is interesting but not the most important part of the paper, which is to determine the role of brk in this system.
The paper then goes on to brk loss and gain of effect studies As the authors admit the loss and gain of effect studies are confusing because they can produce the same phenotypes. I think the issue that is hampering the analysis is that the Gal4 lines being using are too widespread. For example nos-Gal4 is used, but this drives expression in both the GLCs and their derivatives the cysts. There appears to be no brk expression in the GLCs but there does appear to be low level brk expression in the cysts. So a simple hypothesis would be that elimination of brk expression is essential for GLC maintenance and expression is required for differentiation into cysts. Driving brk expression or knocking it out in both the GLCs and the cyst cells cannot address that possibility. Similarly for the somatic cells, the traffic jam-Gal4 may be too widespread to get an understanding of what is going on, in particular when brk appears to be expressed at very different levels in the two key cell types, the CCs and ECs. I'm guessing there are no more specific Gal4 lines that could be used.
One of the major claims of the paper is that brk is directly activating the expression of dpp, which is kind of contradictory to how it behaves in some other tissues, although the primary relationship between dpp and brk in other tissues is that dpp negatively regulates its expression. So it is interesting here that it is show that there is overlap between brk and dpp in the CCs.
However, this is not necessarily that surprising because there is no dpp signaling in the CCs as evidenced by no pMad ab staining. The main evidence that brk is required for dpp expression in the CCs is that knocking down brk results in reduced dpp expression, but extrapolating that to a direct relationship is perhaps a bit too much – brk is apparently required to maintain or promote the differentiation of CCs and so if they aren't there, there won't be any dpp expression. Upregulation of dpp following upregulation of brk expression would make this more convincing, Fig 4A and B are not convincing (and it is impossible to see if brk is actually upregulated in B, although GFP in the reporter deletion is upregulated, brk itself might not be in the brk deletion).

Other comments I think reordering the paper would make it more effective: (1) brk is expressed in the anterior ovary (2) it is required there (3) then do the reporter analysis.
It would have been nice to have shown brk expression after RNAi knock down to confirm the RNA works and to confirm the HCR (what is background?)

Reviewer 3

Advance summary and potential significance to field

This is an interesting study focusing of two interconnected parts. The first focus is on regulatory elements in brinker gene, a key transcription factor in BMP signaling in multiple tissues and stages. The authors study the contribution of a promoter proximal element (PPE) in ovarian regulation of brk and uncover that, similar to its role in the embryo, the PPE serves to coordinate the action of a distant enhancer for brk activation in follicle cells at mid oogenesis. In addition the PPE seems to act as a classical enhancer to regulate brk expression in somatic and germline cells of the germarium. In this context, different regions of the PPE have different effects on transcription with the proximal region dampening the activity of the distal region in most of the cell types. In addition, the authors establish that brk is indeed expressed in the multiple cell types in the germarium, suggesting a role for brk in early oogenesis. By combining genetic analyses with in locus manipulation of regulatory regions, the authors uncover a role for Brk in the regulation of germline stem cell homeostasis. Brk seems to affect both GSC and niche cell fates and such effects seem to be mediated, at least in part, by brk-dependent activation of dpp transcription.
The findings of the first part are interesting but rather preliminary: The PPE was identified before (by the same authors) to mediate coordination of distant enhancers, its role is expanded here by

© 2022. Published by The Company of Biologists under the terms of the Creative Commons Attribution License (https://creativecommons.org/licenses/by/4.0/).

the demonstration of another distant enhancer (brkB) that absolutely depends on this element for activation of brk transcription in posterior follicle cells in mid oogenesis. In this context, it is not clear whether the PPE (given the close proximity to the transcriptional start of the gene would qualify as (part) of the minimal promoter. Connected to that, it is not clear whether the PPE is also involved in integrating the activity of additional enhancers at different stages of development and in different tissues. The second role of the PPE is that of a classical enhancer and it is not new that subfragmentation of such sequences may result in fragments activating different levels of transcription since as repressor and activator binding sequences might be distributed asymmetrically (i.e. the distal part of PPE contains the majority of the activators , while the proximal PPE (damper) the repressors). The probably more interesting part is that this analysis uncovers that brinker is expressed in the germarium and, as shown by the authors, plays a role in the regulation of GSCs (among other cells). While the expression of brinker in somatic cells (CC, TFs and EC) has been reported before (Chen and McKearin), the function of brk in these cells remained elusive. In addition, expression and function of brk in the germline was not only unreported but even excluded (Chen and McKearin, 2003). In this context the authors convincingly show that the gene is indeed active in germline cells (along with all somatic cells of the niche) in a PPE dependent manner. Moreover the effects in GSC numelt seem to be mediated by Brk acting as an activator of Dpp itself, while expression of brk expression positively correlates with the expression of Dpp; This is indeed highly unusual as Dpp and brk are consider mutually exclusive in their expression , mostly because Dpp signaling represses brk transcription in most contexts (including the follicle cells in mid-oogenesis as discussed by the authors). However, the cells that were indeed found to be positive for brk expression (and for dpp transcription) are not necessarily positive for Dpp signaling (pMad): The authors show predominantly expression in the soma and weak expression in the germline- in the second case it is unclear whether this includes GSCs, the only pMad positive cells in the germline. Still it is novel and interesting to see that brk seems to be involved (in contrast to other tissues) in the activation of dpp, an effect that the authors attribute to the transcription factor Engrailed, which is also activated by Brk and has been shown to positively regulate Dpp.
All in all, the study provides several novel findings that will be of significance in the fields of Dpp signaling, transcriptional regulation and stem cells/niche interaction.

Comments for the author

The findings of the first part are interesting but rather preliminary: The PPE was identified before (by the same authors) to mediate coordination of distant enhancers, its role is expanded here by the demonstration of another distant enhancer (brkB) that absolutely depends on this element for activation of brk transcription in posterior follicle cells in mid oogenesis. In this context, it is not clear whether the PPE (given the close proximity to the transcriptional start of the gene would qualify as (part) of the minimal promoter. Connected to that, it is not clear whether the PPE is also involved in integrating the activity of additional enhancers at different stages of development and in different tissues. The second role of the PPE is that of a classical enhancer and it is not new that fragmentation of such sequences may result in subfragments activating different levels of transcription since repressor and activator binding sequences might be distributed asymmetrically (i.e. the distal part of PPE contains the majority of the activators , while the proximal PPE (damper) the repressors). The probably more interesting part is that this analysis uncovers that brinker is expressed in the germarium and, as shown by the authors, plays a role in the regulation of GSCs (among other cells). While the expression of brinker in somatic cells (CC, TFs and EC) has been reported before (Chen and McKearin), the function of brk in these cells remained elusive. In addition, expression and function of brk in the germline was not only unreported but even excluded (Chen and McKearin, 2003). In this context the authors convincingly show that the gene is indeed active in germline cells (along with all somatic cells of the niche) in a PPE dependent manner. Moreover the effects in GSC numelt seem to be mediated by Brk acting as an activator of Dpp itself, while expression of brk expression positively correlates with the expression of Dpp; This is indeed highly unusual as Dpp and brk are consider mutually exclusive in their expression , mostly because Dpp signaling represses brk transcription in most contexts (including the follicle cells in mid-oogenesis as discussed by the authors). However, the cells that were indeed found to be positive for brk expression (and for dpp transcription) are not necessarily positive for Dpp signaling (pMad): The authors show predominantly expression in the soma and weak expression in the germline- in the second case it is unclear whether this includes GSCs, the only pMad positive cells in the germline. Still it is novel and interesting to see that brk seems to be involved (in contrast to

© 2022. Published by The Company of Biologists under the terms of the Creative Commons Attribution License (https://creativecommons.org/licenses/by/4.0/).

other tissues) in the activation of dpp, an effect that the authors attribute to the transcription factor Engrailed, which is also activated by Brk and has been shown to positively regulate Dpp. Specific comments 1.The input of brkB and PPE in the expression of Brk are nicely dissected, however there is some conflict with the functional analyses (besides the discrepancies already spotted by the authors – for example the non-linear behavior of PPE deletions on expression versus function) that needs to be discussed. Extrapolating from the rigid reporter analysis it seems that brkB is (i) exclusively required for PFC expression and (ii) absolutely dependent on the PPE for function. Accordingly, the germarium is not affected upon removal of brkB from the brk locus, yet the females are sterile (probably due to defects in the FCs) while the PPE mutants are not. One would actually expect the effects of the genomic PPE deletion to be either the same or stronger that brkB deletions, given that the PPE is required for both niche expression and PFC expression. At the same time the PPE can support at least low level PFC expression, thus one can expect that the PPE could compensate for brkB loss. Is there an explanation to this? Do the authors have an NF construct lacking brkB to address the exact contribution of brkB in its native context?

2. There are far too many instances of mislabeling of figures and wrong references to figure panels. In reverse there are several cases of “orphan” panels even in main figures. Some of the instances are given in the section below. However the most glaring case is the paragraph starting at line 329. The whole section seems to be a mix-up of panels from previous versions or shifts in the label, references to absent panels of Fig. 4 and S4. In addition, and even if one completely sticks to the text without cross-checking with the primary data of the figures, the short interpretation of the data seem to be swapped (CC to EC and EC to CC transformation). I really tried hard to follow and to allocate the data to the main text but I had to give up!

3. Connected to the above, the structure of the manuscript makes it in part difficult to follow an already complex story. I suggest that a clear separation of the expression data and functional analysis of Brk. I believe it would make more sense to move the expression HCR-based analysis of brk (Fig 3A and B) directly after the data of Fig. 1 as it directly supports the findings of the reporter analysis and then move to the effects of Brk missregulations by manipulations of the locus (Fig 2) or by loss and gain of function analysis (rest of fig 3).
Other comments line 79: Please use inner instead internal germinal sheath for consistency with the nomenclature line 108: Reference (O’Connor, 2006). While this is an excellent review on the generation of Dpp gradients in the early embryo and pupal wing, this is completely unfitting here, as there is not a single mention of Brk in this review. Any review on Dpp gradient read out in larval wing or early embryogenesis would be a better fit here.
Fig, 1H,L, P, N: The expression of the PPE subfragments in late stages (PFC) is not clear. The authors claim that deletion of the distal PPE (1L) results in loss of PFC expression while proximal PPE deletions affect levels but not the pattern in the same cells (1P). This is not really supported by the pictures, at least in the present resolution: expression seems to be lost in both cases. Maybe the authors can present alternative figures or magnifications to support the claim. Similarly, the authors claim that while deletion of the full PPE in the context of the large reporter (1I/J) results in a complete loss of expression in the relevant cells, deletion of either subregion does not affect PFC expression (see also 1F) However reporter expression seems to be reduced upon deletion of the distal PPE (compare 1N to 1E or 1R).
Fig 1 F: Is there any meaning behind the colors (green and red) in some of the +/- used to quantitate expression levels in the table of 1F? In addition I would be convenient to have an extra column for the expression in the germline for completion

Line 261: Unclear: bab1 Gal4 expresses in CCs and TFs thus it would be correct to suggest that the prominent brk expression in CCs (in addition to TFs) does not contribute to GSC homeostasis Line 272: (Lin et al., Fig 2C) Correct panel to refer to would be Fig 1C Line 285: “, the mosaic and missexpression of brk” unclear formulation.
Fig.3: Would be very helpful to include some Gal4 and FRT control panels to have a “WT” pMad distribution for comparison. Quantification panels (3M and 3N should be appropriately cited in the main text of the results section. Also, is there a difference between brk XA and brkKO alleles used in the mosaic analyses?
Line 297 and Fig. S3P: If I interpret the quantification of S3P correctly, then overexpression of brk by bamGal4 does not have a noticeable effect on GSC numbers.
Paragraph starting at line 300: Here interpretation (text) and figure panels do not make sense (panels S2 B, C and L,M). I.e. either the text is correct or the labeling of the panels. I think the

© 2022. Published by The Company of Biologists under the terms of the Creative Commons Attribution License (https://creativecommons.org/licenses/by/4.0/).

text would match the observed phenotypes only if we assume that the panels have been mislabeled and the brk overexpression and brk RNAi are mutually swapped. I hope this is the case otherwise the interpretation of PPEprox deletions on bam expression would be invalid.

First revision

Author response to reviewers' comments

Response to Reviewers

Reviewer 1
General comments:
-The sample size of most of the quantifications is low. Of the 32 genotypes listed in Table S3, only one has an n<20. The MEAN sample size is 13.5+-4.1 (SD). In my opinion, the minimum n to describe accurately a GSC phenotype is 35 germaria, especially considering the variability found in controls. For instance, an increase in sample size is especially important in Fig 2U, that sets the baseline of changes in GSC number for the rest of the paper; and in this case the sample size is between 8 and 11 germaria per genotype. Far too low for the standards in the field. Also, please give the accurate MEAN+-SEM or SD in the text or figure legends, as graphs are not precise enough to know whether a genotype has, e. g., 2.6 or 2.2 GSCs on average.

We have increased our sample size for all of these quantifications. None of our major conclusions were impacted by this higher n, but the confidence was increased and a few of the conditions that were not previously found to be significantly different became significant with the higher counts. We believe that this clarity greatly improves the manuscript and thank the reviewer for their input. All of the means, SDs and specific p-values are included in supplemental Table S2. Further, graphs were altered so that error bars lie on top of points in order to make visual comparison of means between genotypes easier.

-Lines 162-164: In general, I am missing quantification of some of the results reported. This is one of such examples: "The distal region of the PPE alone (PPEdist, Fig. 1A) drives higher levels of expression in the CCs than is observed for the full PPE2kb, and also drives EC expression comparable to that of PPE2kb (Fig. 1F, K, compared to G)". Still, this is documented only with 2 examples shown in fig. 1. If the AUs are to claim that deletion of one of the PPE subunits increases or decreases levels of expression, this should be quantified. The same applies to other deletion or reporter constructs (PPEdist>mCh, etc).

Based on the reviewers' concerns, we have removed any discussion of levels based on the GFP reporters, and use them only to describe expression patterns (i.e. Fig. 1). Because of the diffuse nature of the cytoplasmic GFP and the fact that *brk* is expressed in so many cell types in the germarium we were not able to reliably quantify the levels of the GFP reporters. However, we were able to perform careful quantification of the small reporters (nuclear RFP signal, now found in Fig. 2) and base our discussion of levels on these reporter constructs. In addition, we have quantified the HCR data supporting changes in *brk* as well as *dpp* levels (now found in Figs. 4 & 6). The discussion of these data was also simplified so that it is easier to understand and based on the quantified data.

-Line 194 and others, fig. 2U: pMad labels GSCs and some cystoblasts (Song et al., Development 2004). To use pMad as a proxy for GSC numbers is incorrect. This applies to several instances in the text, including the final Model and large sections of the DIscussion, in which the AUs talk in terms of GSC numbers. GSCs can only be identified accurately if they possess a spectrosome and are found in contact with cap cells.

We appreciate the correction and have changed all references in the text that describe pMad+ niche cells to undifferentiated germ cells (UGCs) instead of GSCs.

© 2022. Published by The Company of Biologists under the terms of the Creative Commons Attribution License (https://creativecommons.org/licenses/by/4.0/).

-Lines 316-327: The hypothesis of brk acting as a positive regulator of Dpp, while plausible according to the data provided by the authors, is contrary to most of the published literature, so it needs to be backed with more solid evidence. The authors simply rely on qualitative HCR stainings (Figure 4). These need to be quantified, and ideally qPCR quantification of Dpp levels in cap cells should be provided as well. This is particularly important, as this is the most interesting finding of the work.

We agree with the reviewer and chose to quantify the HCR data as it is known to be semi-quantitative in nature (Choi et al., 2018). In addition, it facilitated quantification of the cell specificity of the *dpp* and *brk* expression changes that could not be achieved with qPCR. These measurements (i.e. quantification of HCR data in CCs versus non-CCs) can now be found in Figs. 4 and 6.

Specific comments:

-Line 75 and others: Fig 1 calling in the text is confusing. As it stands, the order of citation is 1B, 1C, 1A, 1F, 1G-H, 1D, 1E, 1S-U, 1J, 1V. Please consider rearranging Fig 1 so that panels are cited in the text in an orderly manner.

We have split Fig. 1 into two figures (now Figs. 1 & 2) and have simplified the discussion of cell types in order to display the data more clearly.

-Fig. 1B: What does the darker cell next to the GSCs indicate? It is confusing that it has the same colour as the oocyte of later stages. Oocyte in S2 egg chamber should be at the posterior.

This figure has been altered in accordance with the reviewers comments.

-Figs 1D-E, 1G-R are difficult to see as they are very small (and, in this particular pdf file, resolution is pretty poor). Also, I believe the introduction to TF+cap cells and to Escort cells needs panels with separate channels. For instance, one panel for the tj signal, another for the LamC signal and another one for the brkNFgfp signal.

The figure has been reorganized to make all of the images easier to see.

-Lines76-77: I would not say that a GSC "differentiates" into a CB in physiological conditions. The germarium supports
1) the differentiation of one of the GSC daughters into a CB and 2) the maintenance of the GSC lineage by the other.

Understood. We have addressed this comment in the text.

-Lines 79-81: I would only consider the anterior escort cells as part of the GSC niche, not the rest of the escort cell population, which conform the so-called (albeit I do not like this nomenclature) "differentiation niche".

We have changed to text to specify that it is an anterior set of EC that form the niche. Although we are aware that there are a number of recent studies that have investigated the different populations of the EC in the germarium, for the purposes of the introduction to this study we focus on anterior ECs and therefore to streamline and clarify the text, do not go into depth about further distinctions.

-Line 79: Inner, not interior. AlternatiVEly? Corrected.

-Lines 92-93: Please cite Wilcockson + Ashe, Dev Cell 2019. Added.

-Line 99: Consider "cystoblast and cyst development". There are more relevant papers to refer to the dependence on escort cells of the germline, such as Banisch et al., Dev 2017. Reference included.

© 2022. Published by The Company of Biologists under the terms of the Creative Commons Attribution License (https://creativecommons.org/licenses/by/4.0/).

-Line 112: consider "in this tissue". Changed.

-Line 133: "facilatory" typo? What does this sentence mean? Please explain better in the text the difference between a traditional enhancer and a facilitating(?) one.

This was a typo, it was meant to be "facilitatory" (def: inducing or involved in facilitation). We have changed the text to "facilitate action of other enhancers " to avoid confusion.

-Line 138: a-Spec also labels fusomes, not only spectrosomes. Added text to clarify.
-Lines 140, 141: Please show higher magnifications or larger panels. In their current sizes, it is difficult to distinguish the different cell types. Fig 1 was reorganized to address these issues.

-Lines:143, 144: No specific expression in polar cells can be observed in the current panels. The S10b shown has GFP staining in all of the columnar follicle cells. Why do the AUs state that "brkNFgfp persists in follicle cells throughout egg chamber development, refining to only the posterior follicle cells (PFC) after stage 7"?

By posterior follicle cells we were referring to the columnar follicle cells (CFCs), as opposed to the stretched cells in the anterior of the egg chamber. To avoid confusion, we now refer to these follicle cells exclusively as CFCs.

126-184: I find the description of the different construct tedious and confusing (I have spent literally hours on these lines, going back and forth between the text, fig 1 and fig S1). I would suggest the AUs the following order: 1) brkNFgfp expression to claim a role for brk in the germarium/ovary, 2) brkNFgfp delta PPE to demonstrate the role of the PPE enhancer, 3) brkNFgfp delta PPE prox and distal to suggest the idea of modality of PPE. Then, use the mCHerry reporters to confirm the previous findings and the brkB data to support multimodality. Of course, this changes in the text should be accompanied by the modification of fig 1.

We appreciate the reviewer's feedback and have worked to clarify our presentation. In particular, we split the data from original Fig. 1 into two figures - current Figs. 1 and 2. This allows us to discuss first the requirement for PPE and deletions in the context of large reporters (*brkNFgfp)* with the mutations listed as Reviewer 1 suggested (new Fig. 1). Then we show sufficiency of *PPE* as well as *PPEprox* and *PPEdist* domains (mCherry reporters, as well as the *brkB* reporter) in supporting expression in ovarioles (new Fig. 2). Images of *brkNFgfp* and mCherry reporters in mid-stage egg chambers remain in Fig. S1 as the focus of this work is in the germarium (PPE as enhancer) and to a lesser extent in the late (i.e. stage 10) CFCs (PPE as facilitator, i.e. of *brkB*).

-Line 187: Which "important developmental process" do the AU refer to? Consider "important structure".

To clarify, the text was changed to: "The fact that we observe *brk* expression in cells that comprise the germline stem cell niche (i.e. TF, CCs and ECs) indicates that Brk might play a role in regulating germline homeostasis."

-Line 189: Please refer to Suppl Material for the exact mapping of the deletions giving rise to the delta PPE2kb, delta PPE dist and delta PPEprox alleles. Added call to text for Table S3, which shows exact mapping of deletions.

-Line 202: The increase in round spectrosomes may indicate as well that there are more GSCs giving rise to more cystoblasts, not necessarily that there is a delay in CB differentiation. In my opinion, it is much more telling the (allegedly) lower numbers of fusomes in the experimental germaria that, if properly quantified, could provide evidence to indicate that CB differentiation is truly affected. In addition, in normal conditions GSC differentiation is of little significance.

This is an interesting suggestion but we feel that a more careful analysis of CB differentiation is beyond the scope of the current work. Here we decided to focus on analysis of the PPE, to compare/contrast its role in the ovary with previous work in the embryo, and to highlight that Brk functions both in the soma as well as the germline with readily quantifiable perturbations of UGC number as a readout.

© 2022. Published by The Company of Biologists under the terms of the Creative Commons Attribution License (https://creativecommons.org/licenses/by/4.0/).

-Line236: Please quantify. We have quantified the HCR signal for both *brk* and *dpp* for all genotypes used. See new Fig. 4F, G and Fig. 6G-J, as well as Table S1. In this particular case, the upregulation in delPPEprox is now quantified in the revised Fig. 4.
-Line 258-259: Incorrect. Number of pMad+ cells and number of GSCs are not equal to one another. Addressed. As mentioned above, we now refer to pMad+ cells in the anterior of the germarium as undifferentiated germ cells (UGCs) and no longer refer to them as GSCs.

-Line 248: Incorrect. Bab1-Gal4 is expressed in escort cells too, albeit with lower levels. In fact, it has been used to generate escort cell clones in addition to TF and cap cell ones.

We used GTRACE to lineage-trace expression supported by the *bab1-GAL4* driver. We detect minimal direct expression within the escort cells (see representative image below, RFP driven directly by bab1-GAL4). Because our *bab1-GAL4* FRT experiments did not produce a significant effect on UGC number (previous manuscript) and because TF and CCs do not divide in the adult, we conclude that if there were any low-level *bab1-GAL4* activity in ECs it was not sufficient to generate significant mosaic tissue. Unfortunately the GFP transgenes we used did not permit reliable visualization of clones in germaria (see response to subsequent reviewer comments below for more details on generation of mosaics) and we therefore cannot draw conclusions about the extent of mosaicism resulting from *bab1-GAL4*. To support our analysis of a role for *brk* in the soma we used additional somatic drivers (*c587-GAL4* and *GMR25A11-GAL4* in addition to *tj-GAL4* presented in the original manuscript) all of which support the interpretation that *brk* acts in the ECs to regulate germline homeostasis.

We have removed unpublished data provided for the reviewers in confidence.

-Lines 256 and more: It is essential that the AU show that they can generate mosaic germaria. The ought to identify mutant escort cells with the appropriate markers (i. e., lack of GFP or similar to label mutant cells) and correlate loss of brk function with a phenotype in the germ line (if so).

With our initial approach, we used a cytoplasmic ubi-GFP and intended to make negatively marked clones; however, GFP signal was not readily visible in the germarium. Despite this, the fact that our mosaic experiments matched (non-GAL80ts) misexpression experiments indicate that clones were being generated in the intended germaria cell types. Further we were able to see clones in later egg chamber follicle cells indicating that the GAL4/UAS-FLP/FRT system was working as intended and generating earlier clones even if the marker was not visible. Further, control germaria were of identical genotype to experimental genotypes but lacked UAS-FLP indicating that spontaneous recombination does not occur often enough to significantly affect UGC number. Further, the fact that UGC number trends are opposite in tj-GAL4 and nos-GAL4 experiments and match the misexpression trends suggests that It is also unlikely that leaky UAS-FLP itself could cause non-specific recombination unrelated to GAL4 activity.

For this revised manuscript, we again generated clones with an alternate nuclear ubi-GFP marker. While we were better able to observe this nuclear GFP signal than with the non-NLS clone marker, signal was still not readily discernible in germaria (for example, Rebuttal Fig. 2A, A' below, white arrow). For mosaic experiments using *109-30- GAL4*, large clones could be readily observed in all late-stage egg chambers indicating that the process was efficient and consistent (Rebuttal Fig. 2A, A', white outline). Mutant germaria were difficult to obtain using tj-GAL4 driver and this alternate NLS-ubi-GFP, UAS-FLP line, as that line was unfortunately relatively unhealthy and ovaries from this genotype are already highly perturbed. However in one of the clearest samples, clones are in fact visible in germaria, but only after the intensity of the GFP channel is increased dramatically (Rebuttal Fig. 2B, B' white outline). GFP signal was not visible in the germline with either FRT-marker line examined.

To summarize, despite limited ability to visualize mosaicism in germaria we are confident that GAL4/UAS-FLP clones were being induced due to the facts that (i) clones could be observed in later stage egg chambers , (ii) the GAL4 drivers we focus on (*tj-GAL4* and *nos-GAL4*) are well characterized as driving consistent, distinct domains of expression and (iii) FRT experimental trends in UGC numbers matched those of parallel misexpression experiments in all cases. As these data

© 2022. Published by The Company of Biologists under the terms of the Creative Commons Attribution License (https://creativecommons.org/licenses/by/4.0/).

support our findings from misexpression experiments, we felt it was important to include them to demonstrate that our conclusions are drawn from multiple data sources.

We have removed unpublished data provided for the reviewers in confidence.

As it stands, the experiment in which the AUs look at the consequences for germline development when removing brk from escort cells + cap cells or the germ line is inconclusive.

We have added several new experiments to this revised manuscript that we feel addresses the question of how somatic vs. germline-expressed *brk* influences the germline. Specifically, we have used 3 GAL4 drivers active in the soma of the germarium to discern that EC brk specifically is sufficient to explain the changes in undifferentiated germ cell number observed in PPE mutants. We have also added a second germline driver to support our initial studies with *nos-GAL4*. We have also added experiments using GAL80ts to constrain GAL4 activity to the adult, which allowed us to discern that there were some confounding effects from pupal GAL4 activity that complicated our analysis of results in the original manuscript. See new Figs. 5 and S3. We also quantified *brk* and *dpp* expression in somatic and germline misexpression experiments and these data provide further support such that we now feel that we have a strong model for how somatic and germline *brk* affect GSC homeostasis in the germarium (Fig. 7). Additionally, these improvements to our misexpression approach allowed us to separate germarium *brk*-associated phenotypes from those involved in later follicle cell specification/development (see new Fig. S4). We hope the reviewer will deem the manuscript much improved.

-How come is Fig S3 cited before Fig S2? The text is difficult to follow, not only because the description of the phenotypes rotates from null conditions to delta PPE to delta PPE dist and delta PPEprox, but also because the order of in text figure calling is chaotic.

We have worked to clarify our presentation and hope that the reviewer will find it much improved. In particular, we checked figure calls and hope they are better in line with the text.

-Lines 256 and more: What is the genotype of the flies? How were the clones induced, simply by expressing UAS- FLP under the gal4 drivers, or was tub-Gal80ts also used? This is particularly important in the case of the bab1-Gal4 driver, since TF cells and cap cells do not divide in the adult. And if tub-Gal80ts was not used in conjunction with bab1-Gal4, then the AUs should find escort cell clones when utilizing bab1-gal4. However, they report no phenotypes in the germline. This makes even more compelling labelling the clones.

In the revised analysis, we increased the number of data scored and indeed found that there is a germline phenotype for the *tub-Gal80ts bab1-Gal4 brk* RNAi as well as ectopic expression. We were not aware that the TF cells and cap cells do not divide in the adult, and thank the reviewer for this insight. The clonal analysis using *bab1-Gal4* was removed. To provide additional support for our model (i.e. Brk action in soma affects pMad+ cells in the germline), we have instead used additional somatic drivers to support our conclusion of an EC role for Brk (see Figures 4 and S3). Additionally, we have added a sentence when introducing the FRT approach that more clearly states exactly how clones were generated.

-Line276-277: Are the AUs sure they mean “mislocalisation of the oocyte nucleus”? This is not documented in the figures.

We no longer comment on changes in morphology of the oocyte nucleus. Instead, as further analysis of somatic drivers indicated a shared phenotype between *brk* knockdown with either *tj-GAL4* (with *GAL80*^{TS}) or *109-30-GAL4*, we have revised the figure (now Fig. S4) to focus on this finding.

-Line 283 and more: Have the AUs quantified the decrease in brk levels when using the RNAi construct? Or, for that matter, the increase in levels after overexpressing UAS-brk? This may help understand the controversy of RNAi and overexpression giving the same gain of GSCs phenotype.

© 2022. Published by The Company of Biologists under the terms of the Creative Commons Attribution License (https://creativecommons.org/licenses/by/4.0/).

Both *brk* and *dpp* levels have now been quantified after *brk* misexpression using HCR signal intensity; these data are presented in Figure 6 as well as Table S2. Overexpression of brk using *UAS-brk* is obvious in both the somatic as well as the germline drivers. The *brk* RNAi shows a significant decrease in *brk* levels in the CC in the *tj>brkRNAi* experiment, but not in the *nos-GAL4>brkRNAi*, demonstrating that the drivers are specific to the soma vs. the germline. In the non-CC quantifications of *brk* levels, both the somatic and germline drivers exhibit a slight downward shift in the mean *brk* signal intensity after *brk* RNAi, but the lack of significance for these quantifications is likely due to the fact that we are only decreasing *brk* in a portion of the cell population (i.e. somatic downregulated with *tj-GAL4* and germline normal; or vice versa germline downregulated with *nos-GAL4* and somatic normal).

Further, we repeated our key misexpression experiments in a *GAL80*^{TS} background to determine if pupal GAL4 activity might be confounding our analysis of the adult germarium. Indeed, in the presence of *GAL80*^{TS}, *nos-GAL4* misexpression affects UGC number only when *brk* levels are knocked down (Figure 5J). These experiments are described in detail in the Results section and our proposed model for the relationship between *brk* levels and UGCs in the soma and germline is presented in Figure 7.

-Line 301: I am not sure I would use the Song et al. reference to cite that Bam is necessary and sufficient for GSC differentiation. Consider works from the McKearin lab.

In order to simplify the discussion, we have removed the discussion of *brk* effects on Bam.

-Line 338: Incorrect figure calling. Fig 4N does not exist.

We apologize for making mistakes with figure calls. In this revision, we did our best to make sure these errors were avoided.

-Line 339: How do the AUs know that the DE-cad expression shown in the picture at the germline-escort cell boundaries is in the escort cells? It could equally be in the germline. To claim that overexpression of brk induces higher DE-cad levels in escort cells, the AUs should use an escort cell-specific marker in addition to DE-cad. In addition, DE-cad expression is not only important to keep GSCs in their niche, but also to allow the germline cysts to acquire proper morphology, so the argument put forward by the AUs may not be so.

We have included new images in the supplement (Fig. S6D-H) to illustrate the upregulation of DE-cad that we observe. In the regions highlighted in each of these images the size of the nuclei of the germline vs the follicle cells is sufficient to define the cell types. The cells we are highlighting that are overexpressing En are small and to the outside of the germarium. Included below are images where the ΔPPEprox was crossed with tj>uas-Cherry to mark all of the follicle cells (see Rebuttal Fig. 3). The En and the Cherry signal colocalize and the DE-cad is enriched surrounding many of these cells. There may also be upregulation of DE-cad in the germline but discussion of that is outside of the scope of this manuscript.

We have removed unpublished data provided for the reviewers in confidence.

-Line 346, Fig S2N-R: If one is to compare LamC expression levels in two different germaria, cap cell signal should be similar (assuming that the experimental conditions do not affect levels in the cap cells, of course). And this is not the case in these panels. Please normalize LamC expression with cap cell levels.

In order to simplify the discussion, we have removed mention of LamC expression from the updated manuscript.

-Lines 372-373, Fig. 5B. From the figure, it seems that brk is expressed in hub, CySCs, somatic cells and pre-meiotic 8-16-cell cysts. However, co-stainings with tj and vasa are necessary to confirm the cell types expressing brk. In addition, these co-stainings would help the authors to clarify what

© 2022. Published by The Company of Biologists under the terms of the Creative Commons Attribution License (https://creativecommons.org/licenses/by/4.0/).

they mean with “a subset of somatic cyst cells” (for example, does this mean only early cyst cells?).

To focus the study and appropriately address analysis of the female germline experiments, the testes data were removed.

-Fig. S5: Vasa staining is of very poor quality. FasIII co-staining with GFP is necessary, as it is difficult to distinguish the position of the hub (if it weren´t for the doted circles, it would be impossible). For example, it is not clear if BrkA is expressed in the hub or in some CySCs.

See above; the testes work has been removed.

-Lines 388-391: To address the effects of brk mutants on the male GSCs, the authors simply check pMad levels (Fig S6). There are more direct ways of counting male GSCs (either as Vasa+ in contact with the niche, or as round spectrosomes oriented towards the hub) or to check a possible expansion of CySCs (with Zfh1 staining). Also, please double stain the hub, so that the reader knows where to find the GSCs.
See above; the testes work has been removed.

-Lines391-394: The AUs claim there is an anterior expansion of Engrailed expression in the testis upon brk overexpression (delta PPEprox) and they draw parallelisms with the ovary. However, according to what is shown in Fig S6E-F, both phenotypes are not comparable. While in the ovary, ectopic Engrailed it is expressed in ECs, in the case of the testis it seems as if it is in germline cells, perhaps in 8-cell transient-amplifying cysts and onwards. In fact, this phenotype does not need to be necessarily explained as an expansion of the domain of En expression: it appears to be simply the result of a reduction in the number of early transient-amplifying cysts. Typically, this could be the result of a reduction in GSC proliferation or numbers, causing late cysts to be closer to the niche.

See above; the testes work has been removed.

-Discussion: It is interesting that depending on the Gal4 driver used, brk GOF and LOF show different results. Thus, while using tj GOF and LOF induces opposite phenotypes in terms of GSC numbers (as expected), both GOF and LOF in the germline (utilizing nos-Gal4) cause an increase in GSCs. The AUs may want to mention this in the Discussion.

We have added a summary of phenotypes to the last paragraph of the results, just before the discussion.

-Lines 457-462: some of the mutant phenotypes reported are pleiotropic and indicate “poor cell fitness” of brk mutant cells, raising the question of what are the real effects of eliminating brk in the ovarian niche. This is why labelling the null escort and cap cells in the mosaic germaria is so crucial.

To provide clarity regarding these pleiotropic phenotypes, we (i) incorporated use of GAL80^{TS} to constrain GAL4- supported phenotypes to the adults, (ii) used additional GAL4 somatic drivers, and (iii) focus our results on *brk* function in the ECs and soma (rather than the CCs) because the *bab1-GAL4* data could not be supported with mutant clonal analysis (i.e. as the reviewer pointed out above, CCs and TF cells do not divide in the adult). Using GAL4 drivers with more limited expression domains allowed us to observe that when *brk* RNAi is expressed in the anterior EC (*GMR25A11-GAL4*) there is a loss of UGC, but normal follicle phenotypes in mid-stage egg chambers; while *brk* RNAi using a driver that is in the FSC and their progeny (*109-30-GAL4*) does not cause a loss of UGC but does have perturbed development in later stages. These data points allow us to conclude that these two phenotypes are separable. See Figs. S3 & S4.

-Fig S2F-G: how do the AUs assay cell death? Please show.

We have removed discussion of the late phenotypes associated with the somatic misexpression experiments in order to simplify the discussion. These are areas of interest for future work, but are no longer included here.

© 2022. Published by The Company of Biologists under the terms of the Creative Commons Attribution License (https://creativecommons.org/licenses/by/4.0/).

-Fig3E-H lacks images of tj >+ and nos> + controls, necessary for comparison. The same is true for FigS3. Figure 4 calling in the Discussion is wrong (there are no 4N or 4O panels).

We have added wildtype images to this figure (current Figure 4). Errors in figure calls have been corrected and we apologize for the confusion.

Reviewer 2

Comments for the Author:

Dunipace et al investigate the role of the transcriptional repressor, Brinker (Brk) in the developing ovary of Drosophila, specifically its role in controlling the ability of germline stem cells (GLCs) to both self-renew and differentiate ultimately into an oocyte and support cells. This is the first report on a role for brk in this system and so it is important that this paper does clearly show that brk is important in this regard. However, as the authors themselves indicate, interpreting the data is difficult and a definitive role(s) for brk in this system remains elusive.
We agree that the molecular role of brk may remain unclear; however in this revised manuscript we now more clearly show that brk upregulation in the soma and brk downregulation in the germline lead to an increase in pMad+ undifferentiated germ cells (UGCs). Brk works in multiple cell types comprising soma and germline. Moreover, while in the germline it assumes its canonical role relative to *dpp* signaling (i.e. repressor), in the soma *brk* levels correlate with *dpp*. In addition to these novel insights about how Brk plays multiple roles in the differentiation niche; we also provide insight into the role of a promoter proximal element (PPE) at the *brk* locus that regulates gene expression levels in the ovary.

The paper starts off with reporter analysis to show that brk is expressed in cells in the anterior ovary, high in cap cells (CCs), lower in escort cells (ECs) and possibly not in the germline. This analysis is clear and convincing. Deletion analysis of the relevant enhancer(s) is relevant and good but interpretation is difficult because although these deletions can have opposite effects on brk expression they can have similar cellular phenotypes, i.e. increased number of GLCs, but then have no effect on fertility. As the authors point out, regulation may occur to counteract the earlier affects to allow normal fertility. So this section is interesting but not the most important part of the paper, which is to determine the role of brk in this system.

The paper then goes on to brk loss and gain of effect studies
As the authors admit the loss and gain of effect studies are confusing because they can produce the same phenotypes. I think the issue that is hampering the analysis is that the Gal4 lines being using are too widespread. For example, nos-Gal4 is used, but this drives expression in both the GLCs and their derivatives the cysts. There appears to be no brk expression in the GLCs but there does appear to be low level brk expression in the cysts. So a simple hypothesis would be that elimination of brk expression is essential for GLC maintenance and expression is required for differentiation into cysts. Driving brk expression or knocking it out in both the GLCs and the cyst cells cannot address that possibility. Similarly for the somatic cells, the traffic jam-Gal4 may be too widespread to get an understanding of what is going on, in particular when brk appears to be expressed at very different levels in the two key cell types, the CCs and ECs. I'm guessing there are no more specific Gal4 lines that could be used.

We thank the reviewer for this feedback. Now using GAL80ts in combination with the drivers described in the previous manuscript, nos-Gal4 and tj-Gal4, as well as additional drivers for the soma and germline, we feel that that the results support a model where Brk supports opposite phenotypes in soma and germline: ectopic expression in the soma vs. knock-down in the germline leads to similar increases in UGC number. Specific to this comment, we used the *bam- GAL4* driver, which is expressed in the differentiating cystoblasts, but not in the GSCs, and observed the same effect with an increase of UGCs when *brk* was specifically knocked down in that cell population.

One of the major claims of the paper is that brk is directly activating the expression of dpp, which is kind of contradictory to how it behaves in some other tissues, although the primary relationship between dpp and brk in other tissues is that dpp negatively regulates its expression. So it is interesting here that it is show that there is overlap between brk and dpp in the CCs.

© 2022. Published by The Company of Biologists under the terms of the Creative Commons Attribution License (https://creativecommons.org/licenses/by/4.0/).

Indeed, we see overlap between *brk* and *dpp* in the CCs as well as non-CCs (e.g. ECs). We appreciate the reviewer's interest in these results.

However, this is not necessarily that surprising because there is no dpp signaling in the CCs as evidenced by no pMad ab staining. The main evidence that brk is required for dpp expression in the CCs is that knocking down brk results in reduced dpp expression, but extrapolating that to a direct relationship is perhaps a bit too much – brk is apparently required to maintain or promote the differentiation of CCs and so if they aren't there, there won't be any dpp expression. Upregulation of dpp following upregulation of brk expression would make this more convincing, Fig 4A and B are not convincing (and it is impossible to see if brk is actually upregulated in B, although GFP in the reporter deletion is upregulated, brk itself might not be in the brk deletion).

We appreciate the feedback. We quantified the HCR signal for *dpp* and *brk* in CCs as well as germarium non-CCs and this data within Fig. 4 and 6 now supports the view that ectopic brk leads to an increase in dpp levels within CCs as well as non-CCs.

Other comments
I think reordering the paper would make it more effective: (1) brk is expressed in the anterior ovary (2) it is required there (3) then do the reporter analysis.

We appreciate the reviewer's feedback. We did split the data contained in figure 1 and hope that the presentation is now more clear. We discuss that (1) brk is expressed in the anterior ovary in a manner influenced by the promoter proximal element (PPE); (2) the reporter analysis suggests that the PPE regulates brk levels in the germarium; and (3) PPE mutants show changes in brk levels and germarium phenotypes that include an increase in pMad+ undifferentiated germ cells (UGCs).

It would have been nice to have shown brk expression after RNAi knock down to confirm the RNA works and to confirm the HCR (what is background?)

This is now shown in Fig. 6 and results are quantified (see Table S2).

Reviewer 3

Comments for the Author:

The findings of the first part are interesting but rather preliminary: The PPE was identified before (by the same authors) to mediate coordination of distant enhancers, its role is expanded here by the demonstration of another distant enhancer (brkB) that absolutely depends on this element for activation of brk transcription in posterior follicle cells in mid oogenesis. In this context, it is not clear whether the PPE (given the close proximity to the transcriptional start of the gene would qualify as (part) of the minimal promoter.

The full 2kbPPE deletion does not remove the minimal promoter that we defined in our previous paper and showed functions in the embryo (Dunipace, 2013). Furthermore, the PPEprox deletion, which has the same 3' break point as the 2kbPPE deletion, is able to support expression in the ovary. Lastly, the RNAseq data available through FlyBase indicates that the transcript for both the embryo and the ovary have coincident start points, making it unlikely that an alternative promoter would be contained within PPEdist, more than 1kb upstream of the mapped transcript. Moreover, the PPEdist deletion, similar to the PPEprox deletion, is also able to support expression (e.g. Fig. 1H-K').

Connected to that, it is not clear whether the PPE is also involved in integrating the activity of additional enhancers at different stages of development and in different tissues.

Although we agree this is an interesting direction, it is beyond the scope of this work to look at other stages or tissues. Our first paper showed that brk expression in the wing disc and late embryo are not entirely dependent on the PPE, and in this study we show that the PPE is required for expression

© 2022. Published by The Company of Biologists under the terms of the Creative Commons Attribution License (https://creativecommons.org/licenses/by/4.0/).

in the entire ovary, including supporting expression of *brkB* enhancer in stage10 egg chambers. There are at least two other uncharacterized enhancers that are active in the ovary that require the PPE, but we are not showing this data as we prefer to focus on showing the effect on the published enhancer.

The second role of the PPE is that of a classical enhancer and it is not new that fragmentation of such sequences may result in subfragments activating different levels of transcription since repressor and activator binding sequences might be distributed asymmetrically (i.e. the distal part of PPE contains the majority of the activators, while the proximal PPE (damper) the repressors). The probably more interesting part is that this analysis uncovers that brinker is expressed in the germarium and, as shown by the authors, plays a role in the regulation of GSCs (among other cells). While the expression of brinker in somatic cells (CC, TFs and EC) has been reported before (Chen and McKearin), the function of brk in these cells remained elusive. In addition, expression and function of brk in the germline was not only unreported but even excluded (Chen and McKearin, 2003). In this context the authors convincingly show that the gene is indeed active in germline cells (along with all somatic cells of the niche) in a PPE dependent manner.

We are glad that the reviewer appreciates our demonstration that brk is expressed in the germline and plays a role in the regulation of undifferentiated germ cells (UGCs). We also emphasize that previous studies showed that the PPE itself did not drive gene expression in the embryo but influenced action of long-distance enhancers; in contrast to the current study in the germarium/ovary that shows the PPE functions also as an enhancer in addition to supporting long-distance action of the *brkB* enhancer.

Moreover the effects in GSC numeIt (sic number) seem to be mediated by Brk acting as an activator of Dpp itself, while expression of brk expression positively correlates with the expression of Dpp; This is indeed highly unusual as Dpp and brk are consider mutually exclusive in their expression, mostly because Dpp signaling represses brk transcription in most contexts (including the follicle cells in mid-oogenesis as discussed by the authors). However, the cells that were indeed found to be positive for brk expression (and for dpp transcription) are not necessarily positive for Dpp signaling (pMad): The authors show predominantly expression in the soma and weak expression in the germline- in the second case it is unclear whether this includes GSCs, the only pMad positive cells in the germline.
We do not observe expression of *brk* in the GSCs but, as stated in the text, it is challenging to definitively assign an HCR spot to a specific cell. In the niche, all of the cells are closely spaced and there are cytoplasmic extensions of the ECs into the germline and from the germline into the follicle cells. Our impression from looking at many germarium is that *brk* is not expressed in the pMad positive cells. As the reviewer states, this is as would be expected in other systems but, as the current tools we have do not allow us to say this with certainty (i.e. we cannot separate expression associated with somatic cell protrusions from germ cells), we have chosen not to discuss it in the text. It is still remarkable, however, that we can show that *brk* and *dpp* are coexpressed in the soma as it has also been shown that Brk can directly repress *dpp* in other tissues. In this instance within the germarium soma, not only are the genes coexpressed, but *brk* can positively regulate *dpp* expression.

Still it is novel and interesting to see that brk seems to be involved (in contrast to other tissues) in the activation of dpp, an effect that the authors attribute to the transcription factor Engrailed, which is also activated by Brk and has been shown to positively regulate Dpp.

We appreciate the reviewer's positive feedback.

Specific comments
1. The input of brkB and PPE in the expression of Brk are nicely dissected, however there is some conflict with the functional analyses (besides the discrepancies already spotted by the authors - for example the non-linear behavior of PPE deletions on expression versus function) that needs to be discussed. Extrapolating from the rigid reporter analysis it seems that brkB is (i) exclusively required for PFC expression and (ii) absolutely dependent on the PPE for function. Accordingly, the germarium is not affected upon removal of brkB from the brk locus, yet the females are sterile (probably due to defects in the FCs) while the PPE mutants are not. One would actually expect the effects of the genomic PPE deletion to be either the same or stronger that brkB deletions, given

© 2022. Published by The Company of Biologists under the terms of the Creative Commons Attribution License (https://creativecommons.org/licenses/by/4.0/).

that the PPE is required for both niche expression and PFC expression. At the same time the PPE can support at least low level PFC expression, thus one can expect that the PPE could compensate for brkB loss. Is there an explanation to this? Do the authors have an NF construct lacking brkB to address the exact contribution of brkB in its native context?

The phenotype of the *brkB* deletion is not inconsistent with our current findings. We think that the reviewer misunderstood the description of the different CRISPR deletions in context with the reporter constructs; so we have worked to increase clarity of our presentation. The large reporter construct, which indicates that *brkB* requires the *PPE*, is the deletion of the full 2kb PPE. The phenotypes associated with the CRISPR/Cas9 generated mutant deleting this region (i.e. *Δ2kbPPE*) are much more severe than those of the *ΔbrkB* CRISPR mutant. The *2kbPPE* deletion is largely lethal (with only a few escapers per generation) and completely sterile, while the *ΔbrkB* CRISPR mutants are non-lethal but sterile. We will try to make this more clear in the text. The function of Brk in later egg chamber development, which is primarily supported by *brkB*, is outside of the scope of this paper but is an interesting future direction.

2. There are far too many instances of mislabeling of figures and wrong references to figure panels. In reverse there are several cases of “orphan” panels even in main figures. Some of the instances are given in the section below. However the most glaring case is the paragraph starting at line 329. The whole section seems to be a mix-up of panels from previous versions or shifts in the label, references to absent panels of Fig. 4 and S4. In addition, and even if one completely sticks to the text without cross-checking with the primary data of the figures, the short interpretation of the data seem to be swapped (CC to EC and EC to CC transformation). I really tried hard to follow and to allocate the data to the main text but I had to give up!

We apologize for these errors that stem from last minute edits of the original manuscript. This time around, we spent extra time double-checking the figure calls so, hopefully, the presentation will be more clear.

3. Connected to the above, the structure of the manuscript makes it in part difficult to follow an already complex story. I suggest that a clear separation of the expression data and functional analysis of Brk. I believe it would make more sense to move the expression HCR-based analysis of brk (Fig 3A and B) directly after the data of Fig. 1 as it directly supports the findings of the reporter analysis and then move to the effects of Brk missregulations by manipulations of the locus (Fig 2) or by loss and gain of function analysis (rest of fig 3).

As mentioned above in the response to reviewer 2, we have worked on the presentation of our data. We split the data contained in Fig. 1 and hope that the presentation is now more clear. We discuss that (1) *brk* is expressed in the anterior ovary in a manner influenced by the promoter proximal element (PPE); (2) the reporter analysis suggests that the PPE regulates *brk* levels in the germarium; and (3) PPE mutants show changes in *brk* levels and germarium phenotypes that include an increase in pMad+ undifferentiated germ cells (UGCs). The HCR data relating to *brk* and *dpp* levels was moved to the top of Fig. 4, following analysis of UGC phenotypes associated with mutants.

Other comments
line 79: Please use inner instead internal germinal sheath for consistency with the nomenclature

Corrected.

line 108: Reference (O’Connor, 2006). While this is an excellent review on the generation of Dpp gradients in the early embryo and pupal wing, this is completely unfitting here, as there is not a single mention of Brk in this review. Any review on Dpp gradient read out in larval wing or early embryogenesis would be a better fit here.

We have added two other reviews to the citation, although we have left the O’Connor review as we find this one of the best summaries of the “other mechanisms” that help shape the Dpp gradient.

Fig, 1H,L, P, N: The expression of the PPE subfragments in late stages (PFC) is not clear. The

© 2022. Published by The Company of Biologists under the terms of the Creative Commons Attribution License (https://creativecommons.org/licenses/by/4.0/).

authors claim that deletion of the distal PPE (1L) results in loss of PFC expression while proximal PPE deletions affect levels but not the pattern in the same cells (1P). This is not really supported by the pictures, at least in the present resolution: expression seems to be lost in both cases. Maybe the authors can present alternative figures or magnifications to support the claim. Similarly, the authors claim that while deletion of the full PPE in the context of the large reporter (1I/J) results in a complete loss of expression in the relevant cells, deletion of either subregion does not affect PFC expression (see also 1F) However reporter expression seems to be reduced upon deletion of the distal PPE (compare 1N to 1E or 1R).

We have split Figure 1 into two figures so that these images can be displayed more clearly. For the PFC (now referred to as columnar follicle cells (CFC)), there is an inset to highlight the expression (Fig. 2 B, D, F). We hope that it is now clear that there is low level expression driven in the CFC by the *PPE2kb* as well as the *PPEprox*. The panels that the reviewer is referring to are the direct reporters driving mCherry and do not represent deletions of these elements. We did not quantify the GFP reporters, and are therefore not making any distinctions regarding levels of expression for the deletions from these large reporter constructs.

Fig 1 F: Is there any meaning behind the colors (green and red) in some of the +/- used to quantitate expression levels in the table of 1F? In addition I would be convenient to have an extra column for the expression in the germline for completion

Instead of using +/- to designate expression levels, we have added a quantitative analysis based on the output of reporters (Fig. 2G, H). We are unable to reliably detect reporter expression in the germline cells in the germarium, possibly due to the fact that our reporter uses a hsp70 promoter which has been shown to be affected by piRNAs in the germline (Deluca and Spradling, 2018). Instead, our assessment of germline *brk* expression is now based on assessment of HCR signal (Fig. 4) and on demonstration of a genetic requirement there for *brk* expression to maintain germline homeostasis (Fig. 5).

Line 261: Unclear: bab1 Gal4 expresses in CCs and TFs thus it would be correct to suggest that the prominent brk expression in CCs (in addition to TFs) does not contribute to GSC homeostasis.

The experiments using *bab1-GAL4* were inconclusive. The misexpression experiments (both *UAS-brk* and *brk* RNAi) using this driver showed an increase in UGC number and because these cells do not divide in the adult the clonal analyses were not able to confirm these results (Fig. S3). Based on the data from all of the other somatic cell drivers there is strong evidence that *brk* overexpression in the anterior EC is sufficient to cause an increase in UGC number, but we can not rule out a role for *brk* in the CC and TF at this time.

Line 272: (Lin et al., Fig 2C) Correct panel to refer to would be Fig 1C

We apologize for this error and have tried to avoid any errors in figure calls in this revised manuscript.

Line 285: ", the mosaic and missexpression of brk" unclear formulation.
The text has been substantially revised, and we have clarified in the text the idea that the GAL4 system was used to assay knockdown and ectopic expression.

Fig.3: Would be very helpful to include some Gal4 and FRT control panels to have a "WT" pMad distribution for comparison. Quantification panels (3M and 3N should be appropriately cited in the main text of the results section. Also, is there a difference between brk XA and brkKO alleles used in the mosaic analyses?

We have added wildtype images to Figure 5B,G for comparison and ensured that all figure panels are cited in text. Both XA and KO are null alleles [see Table S1, (Campbell and Tomlinson, 1999; Upadhyai and Campbell, 2013)].

Line 297 and Fig. S3P: If I interpret the quantification of S3P correctly, then overexpression of brk by bamGal4 does not have a noticeable effect on GSC numbers.

© 2022. Published by The Company of Biologists under the terms of the Creative Commons Attribution License (https://creativecommons.org/licenses/by/4.0/).

New data with *bam-GAL4* does show a significant increase in UGC number from *bam>brkRNAi* but not from overexpression (Fig. S3). This is in line with the *nos-GAL4, GAL80^{TS}* experiments in which *brk* RNAi but not ectopic expression in the germline was associated with increased UGC number.

Paragraph starting at line 300: Here interpretation (text) and figure panels do not make sense (panels S2 B, C and L,M). I.e. either the text is correct or the labeling of the panels. I think the text would match the observed phenotypes only if we assume that the panels have been mislabeled and the brk overexpression and brk RNAi are mutually swapped. I hope this is the case otherwise the interpretation of PPEprox deletions on bam expression would be invalid.
Reviewer 3 is correct; the panels were indeed mislabeled whereas our interpretation in the discussion/text matched the intended data. However, these data are no longer included in the revised manuscript. Nevertheless, we apologize for the error.

Second decision letter

MS ID#: DEVELOP/2021/199890

MS TITLE: brinker levels regulated by a promoter proximal element support germline stem cell homeostasis

AUTHORS: Leslie Dunipace, Susan Newcomb, and Angelike Stathopoulos

I have now received all the referees reports on the above manuscript, and have reached a decision. The referees' comments are appended below, or you can access them online: please go to BenchPress and click on the 'Manuscripts with Decisions' queue in the Author Area.

The overall evaluation is positive and we would like to publish a revised manuscript in Development, provided that the referees' comments can be satisfactorily addressed. Please attend to all of the reviewers' comments in your revised manuscript and detail them in your point-by-point response. If you do not agree with any of their criticisms or suggestions explain clearly why this is so. I do urge you to consider especially the points about referring to some of the cells under study as “undifferentiated,”¤ which I agree is important to explain in a more nuanced way in this manuscript.

We are aware that you may currently be unable to access the lab to undertake experimental revisions. If it would be helpful, we encourage you to contact us to discuss your revision in greater detail. Please send us a point-by-point response indicating where you are able to address concerns raised (either experimentally or by changes to the text) and where you will not be able to do so within the normal timeframe of a revision. We will then provide further guidance. Please also note that we are happy to extend revision timeframes as necessary.

Reviewer 1

Advance summary and potential significance to field

Not necessary. This is a Second submission.

Comments for the author

In this revised version, the AUs have made a clear effort in order to improve the quality and clarity of the MS.
While some of the results cannot be fully explained in view of the AUs model, I believe the MS is now a solid report dealing with an important problem in stem cell niche regulation: the role of brk and its relationship with dpp signaling in the ovarian GSC niche.

© 2022. Published by The Company of Biologists under the terms of the Creative Commons Attribution License (https://creativecommons.org/licenses/by/4.0/).

Most of my criticisms have been dealt with. Writing my first review took a great deal of my time and I am happy to see that the end result -after a significant effort by the AUs too- is a much-improved MS. I ought to thank the AUs for their constructive attitude towards my original comments. Still, I have some reflections for the AUs which I hope are useful:

- My main comment is that I do not like the UGC (undifferentiated germ cells) term. By definition, a cystoblast is a GSC daughter that has entered differentiation, i. e., it now expresses the differentiation marker bam (and others) and will divide 4 times to make the 16-cell cyst. I would not call a CB undifferentiated. In fact, the AUs refer to “differentiating CBs” in line 143. I would suggest the AUs simply refer to pMad+-cells throughout the MS + figs + legends.
- Line 146: The Wieschaus + Szabad citation to refer to pMad expression in GSCs and CBs may require your attention.
- Lines 153-155: “We also observed that the number of rounded spectrosomes, which mark UGCs and cystoblasts, increased correspondingly with UGC number indicating that these changes represent a delay in the differentiation of UGCs and cystoblasts into more mature cysts”. I do not understand this sentence. If the so-called UGCs are identified by the presence of a spectrosome, then the higher the number of spectrosome-containing cells, the larger the UGC numbers, and viceversa.
- Line 154: UGCs should be GSCs.
- Line 157: I believe I mentioned this in my previous review. The “effects on differentiation” mentioned by the AUs have not been proven in the MS. The extra spectrosomes observed in some of the experimental conditions may be a consequence of impaired differentiation or due to enhanced proliferation and/or increased niche size/activity (and see Fig 6K-P).
- Lines 158-159: The GSCs, present in region 1, are not differentiating cells.
- Lines 163-164: Fig 3H, L, P may have tumorous expansions but a Vasa staining is not sufficient to show them. Either the AUs use an anti-Hts or anti-a-Spectrin staining or the tumorous expansions are very difficult to detect.
- Lines 252-253: Confusing. I would suggest “using either brkDelta prox or UAS-brk with tj-Gal4…”.
- Lines 253-254: In my opinion, there is a significant difference in the tub-Gal80ts experiments. tj + ts>brk RNAi does not reduce Pmad numbers, whereas tj>brk RNAi does.
- Lines 335-336: bam RNA is expressed in CBs and in 2-, 4- and 8- cell cysts (albeit weakly in the later);

Bam protein is detected in CBs, 2-, 4- and strongly in 8-cell cysts. bam-Gal4 is expressed in CBs and, like the RNA and protein, in dividing cysts.

Second revision

Author response to reviewers' comments

Reviewer 1 Advance Summary and Potential Significance to Field:
Not necessary. This is a Second submission.

Reviewer 1 Comments for the Author:
In this revised version, the AUs have made a clear effort in order to improve the quality and clarity of the MS. While some of the results cannot be fully explained in view of the AUs model, I believe the MS is now a solid report dealing with an important problem in stem cell niche regulation: the role of brk and its relationship with dpp signaling in the ovarian GSC niche. Most of my criticisms have been dealt with. Writing my first review took a great deal of my time and I am happy to see that the end result -after a significant effort by the AUs too- is a much-improved MS. I ought to thank the AUs for their constructive attitude towards my original comments. Still, I have some reflections for the AUs which I hope are useful:

-My main comment is that I do not like the UGC (undifferentiated germ cells) term. By definition, a cystoblast is a GSC daughter that has entered differentiation, i. e., it now expresses the differentiation marker bam (and others) and will divide 4 times to make the 16-cell cyst. I would not call a CB undifferentiated. In fact, the AUs refer to “differentiating CBs” in line 143. I would suggest the AUs simply refer to pMad+-cells throughout the MS + figs + legends.

© 2022. Published by The Company of Biologists under the terms of the Creative Commons Attribution License (https://creativecommons.org/licenses/by/4.0/).

We appreciate the reviewers concerns and have changed all references from UGC to “pMad+ cell” in the text and associated figures. Only Figure 3U required updating.

-Line 146: The Wieschaus + Szabad citation to refer to pMad expression in GSCs and CBs may require your attention.

We agree and have changed the citation to: *Song* et al.*, 2004*

-Lines 153-155: “We also observed that the number of rounded spectrosomes, which mark UGCs and cystoblasts, increased correspondingly with UGC number indicating that these changes represent a delay in the differentiation of UGCs and cystoblasts into more mature cysts”. I do not understand this sentence. If the so-called UGCs are identified by the presence of a spectrosome, then the higher the number of spectrosome-containing cells, the larger the UGC numbers, and viceversa.

We have changed it to:
“We also observed that the number of rounded spectrosomes, which mark GSCs and cystoblasts, increased correspondingly with the pMad+ cell number confirming that these changes represent a delay in the differentiation of GSCs and cystoblasts into more mature cysts”

-Line 154: UGCs should be GSCs. Changed

-Line 157: I believe I mentioned this in my previous review. The “effects on differentiation” mentioned by the AUs have not been proven in the MS. The extra spectrosomes observed in some of the experimental conditions may be a consequence of impaired differentiation or due to enhanced proliferation and/or increased niche size/activity (and see Fig 6K-P).
Changed to:
“These observations of effects on germline homeostasis in the niche are further supported by our findings of corresponding changes in overall morphology of the germline in mutant germaria”

-Lines 158-159: The GSCs, present in region 1, are not differentiating cells. Changed.

-Lines 163-164: Fig 3H, L, P may have tumorous expansions but a Vasa staining is not sufficient to show them. Either the AUs use an anti-Hts or anti-a-Spectrin staining or the tumorous expansions are very difficult to detect.

We believe the changes in organization are visible in the images shown using the Vasa staining. Nevertheless, these also were costained with a-Spectrin (as the reviewer suggests – for all genotypes, shown in panels 3B, F,J, N, and R).

-Lines 252-253: Confusing. I would suggest “using either brkDelta prox or UAS-brk with tj-Gal4…”.

The text currently reads: “using either *UAS-brk* or *brk*$^{\Delta PPEprox}$ with the *tj-GAL4*” . We chose to keep the text as written. We are creating clones of *PPEprox* deletion and so we think it might be helpful to keep it this way so the reader has a chance to realize this fact.

-Lines 253-254: In my opinion, there is a significant difference in the tub-Gal80ts experiments. tj + ts>brk RNAi does not reduce Pmad numbers, whereas tj>brk RNAi does.

We agree that there is a significant difference with the knockdown experiments between when using the Gal80ts, and we discuss those changes in the following paragraph. Here we are specifically discussing the overexpression.

-Lines 335-336: bam RNA is expressed in CBs and in 2-, 4- and 8- cell cysts (albeit weakly in the later); Bam protein is detected in CBs, 2-, 4- and strongly in 8-cell cysts. bam-Gal4 is expressed in CBs and, like the RNA and protein, in dividing cysts.

Changed in the text.

© 2022. Published by The Company of Biologists under the terms of the Creative Commons Attribution License (https://creativecommons.org/licenses/by/4.0/).

Reviewer 3 Advance Summary and Potential Significance to Field:

Reviewer 3 Comments for the Author:
In the revised version, the authors put some effort to improve the quality of the manuscript by (i) providing quantifications, (ii) reducing the complexity and (iii) correcting the obvious panel assembly and figure calling mistaces. I still find that the paper would benefit from drastic restructuring (as also suggested by one of the other reviewers): Starting with the "unbiased" expression analysis, then moving to the reporters to identify the responsible elements and finally addressing the function of brinker both by genetics (RNAi/LOF/overexpression) and by in locus manipulation of the identified regulatory regions.

In the previous revised version, we did indeed reorganize the manuscript. It was a drastic restructuring and we feel that the data are organized in a logical fashion that supports our telling of a cogent biological story in this submission.

All in all, the study provides evidence that brk is expressed in many different cell types of the adult germarium (and, as suggested by some experiments, probably also at earlier developmental stages) and contributes to its function. The authors bravely used a number of approaches to dissect Brinker function in the different cell types, which in their sum suggest a very complex and cell-type specific role of Brinker in the regulation of germarium function. The interpretation of the results is often based on cumulative evidence and suffers from the absence of tools that allow specific and acute manipulation of Brinker in a single cell type of the germarium. Nevertheless, the results make a point that somatic Brk is co-expressed with Dpp and positively affects BMP signaling in the germline by positively regulating Dpp expression. This is in stark contrast with the otherwise negative effect of Brk on BMP signaling either at the level of Dpp expression or at the level of Dpp target gene activation.

Minor corrections:
Line 96: …; while expression there is normal in brkNFgfp-ΔPPEprox (Figs. S1C and 1K'),…Rephrased.

Line 161: … region 2a contains the 16-cell cysts and 2B the same

Change 2B into 2b. Changed

<u>Third decision letter</u>

MS ID#: DEVELOP/2021/199890

MS TITLE: brinker levels regulated by a promoter proximal element support germline stem cell homeostasis

AUTHORS: Leslie Dunipace, Susan Newcomb, and Angelike Stathopoulos
ARTICLE TYPE: Research Article

I am happy to tell you that your manuscript has been accepted for publication in Development, pending our standard ethics checks.

© 2022. Published by The Company of Biologists under the terms of the Creative Commons Attribution License (https://creativecommons.org/licenses/by/4.0/).
